# Functional and Structural Characterization of a Receptor-Like Kinase Involved in Germination and Cell Expansion in *Arabidopsis*

**DOI:** 10.3389/fpls.2017.01999

**Published:** 2017-11-22

**Authors:** Zhen Wu, Shan Liang, Wen Song, Guangzhong Lin, Weiguang Wang, Heqiao Zhang, Zhifu Han, Jijie Chai

**Affiliations:** ^1^School of Life Sciences, Innovation Center for Structural Biology, Tsinghua-Peking Joint Center for Life Sciences, Tsinghua University, Beijing, China; ^2^School of Food and Chemical Engineering, Beijing Technology and Business University, Beijing, China; ^3^Max-Planck Institute for Plant Breeding Research, Cologne, Germany; ^4^Institute of Biochemistry, University of Cologne, Cologne, Germany; ^5^School of Life Scienses and Thechology, Shanghai Tech University, Shanghai, China

**Keywords:** GRACE, LRR-RLKs, cell expansion, seed germination, Island domain

## Abstract

Leucine-rich repeat receptor-like kinases (LRR-RLKs) are widespread in different plant species and play important roles in growth and development. Germination inhibition is vital for the completion of seed maturation and cell expansion is a fundamental cellular process driving plant growth. Here, we report genetic and structural characterizations of a functionally uncharacterized LRR-RLK, named GRACE (Germination Repression and Cell Expansion receptor-like kinase). Overexpression of *GRACE* in *Arabidopsis* exhibited delayed germination, enlarged cotyledons, rosette leaves and stubbier petioles. Conversely, these phenotypes were reversed in the T-DNA insertion knock-down mutant *grace-1* plants. A crystal structure of the extracellular domain of GRACE (GRACE-LRR) determined at the resolution of 3.0 Å revealed that GRACE-LRR assumed a right-handed super-helical structure with an island domain (ID). Structural comparison showed that structure of the ID in GRACE-LRR is strikingly different from those observed in other LRR-RLKs. This structural observation implies that GRACE might perceive a new ligand for signaling. Collectively, our data support roles of *GRACE* in repressing seed germination and promoting cell expansion of *Arabidopsis*, presumably by perception of unknown ligand(s).

## Introduction

As sessile organisms, plants develop powerful signal communication systems to survive in the constantly changed environment. Membrane-localized receptor-like kinases (RLKs) are important to perceive signals from highly dynamic internal growing processes and various environmental conditions, thus transmitting them into cell to regulate growth, development, defense processes, and abiotic/biotic stresses (Li and Tax, 2013). LRR-RLKs are the largest and well-studied subfamily of RLKs in *Arabidopsis* (Shiu and Bleecker, 2001a,b). However, functions for most of the LRR-RLKs are still elusive (Wu et al., 2016).

LRR-RLKs have an extracellular LRR region typically responsible for signal recognition, a single-passing transmembrane region, and an intracellular serine/threonine kinase domain for signal initiation (Kobe and Kajava, 2001; Botos et al., 2011; Li and Tax, 2013; Zhang and Thomma, 2013). LRR-RLKs fall into 15 subfamilies with highly diversified functions (Shiu and Bleecker, 2001a,b). The well characterized LRR-RLKs include brassinosteroid insensitive 1 (BRI1) (Clouse et al., 1996; Li and Chory, 1997; He et al., 2000; Kinoshita et al., 2005), phytosulfokin receptor 1/2 (PSKR1/2) (Matsubayashi et al., 2002, 2006; Shinohara et al., 2007), excess microsporocytes1 (EMS1) (Zhao et al., 2002; Jia et al., 2008) that play critical roles in plant growth and development. Some LRR-RLKs contain an island domain (ID) or a non-LRR region interrupting their LRRs solenoid (Matsushima et al., 2009). IDs from several LRR-RLKs have been shown important for ligand recognition and protein-protein interaction (Hothorn et al., 2011; She et al., 2011; Liu et al., 2013; Wang et al., 2015).

Plant growth and development are driven by a series of physiological processes, among which cell expansion and seed germination are two fundamental ones. Germination, incorporating up-take of water and embryonic axis elongation, is the first step of growth after seed dormancy. Several plant hormones were shown to play important roles in seed germination (Karssen et al., 1983; Finch-Savage and Leubner-Metzger, 2006). Cell expansion mainly contributes to morphological growth of organs and tissues. Different fine-tune pathways maintaining appropriate cell expansion rate and direction have been identified (Kinoshita and Shimazaki, 1999; Takahashi et al., 2012; Haruta et al., 2014). Recently, several RLK genes have been reported to participate in these processes, such as RLK7 (Pitorre et al., 2010) and FER (Haruta et al., 2014), involved in regulating germination or cell expansion in *Arabidopsis*, respectively.

To screen visible phenotypes of the functionally uncharacterized members of LRR X subfamily (Shiu and Bleecker, 2001a,b), we studied the expression pattern, identified T-DNA insertion mutants and generated overexpressing lines of them. Probably due to gene redundancy, only a few of them showed obvious differences from wild type Col-0. In this study, an ID-containing RLK (named GRACE) was selected for further analysis. *GRACE* encodes a protein localized on the plasma membrane of various organs, especially seeds and rosette leaves. Overexpression lines of GRACE present delayed germination, enlarged cotyledons, expanded rosette leaves and stubbier petioles compared to Col-0, oppositely to the *grace-1*. The structural study of GRACE reveals that the extracellular domain of GRACE resembles into a super-helical solenoid structure with a separated island domain (ID) anchoring on its concave surface. GRACE-ID is a new folding that distinct from currently reported LRR-IDs. Taken together, the functional and structural data imply that GRACE may function as a receptor of a novel potential signal to negatively regulate germination and positively regulate cell expansion in *Arabidopsis*.

## Results

### *GRACE* is mainly expressed in seeds and leaves and encodes a membrane protein

**Figure 1:**
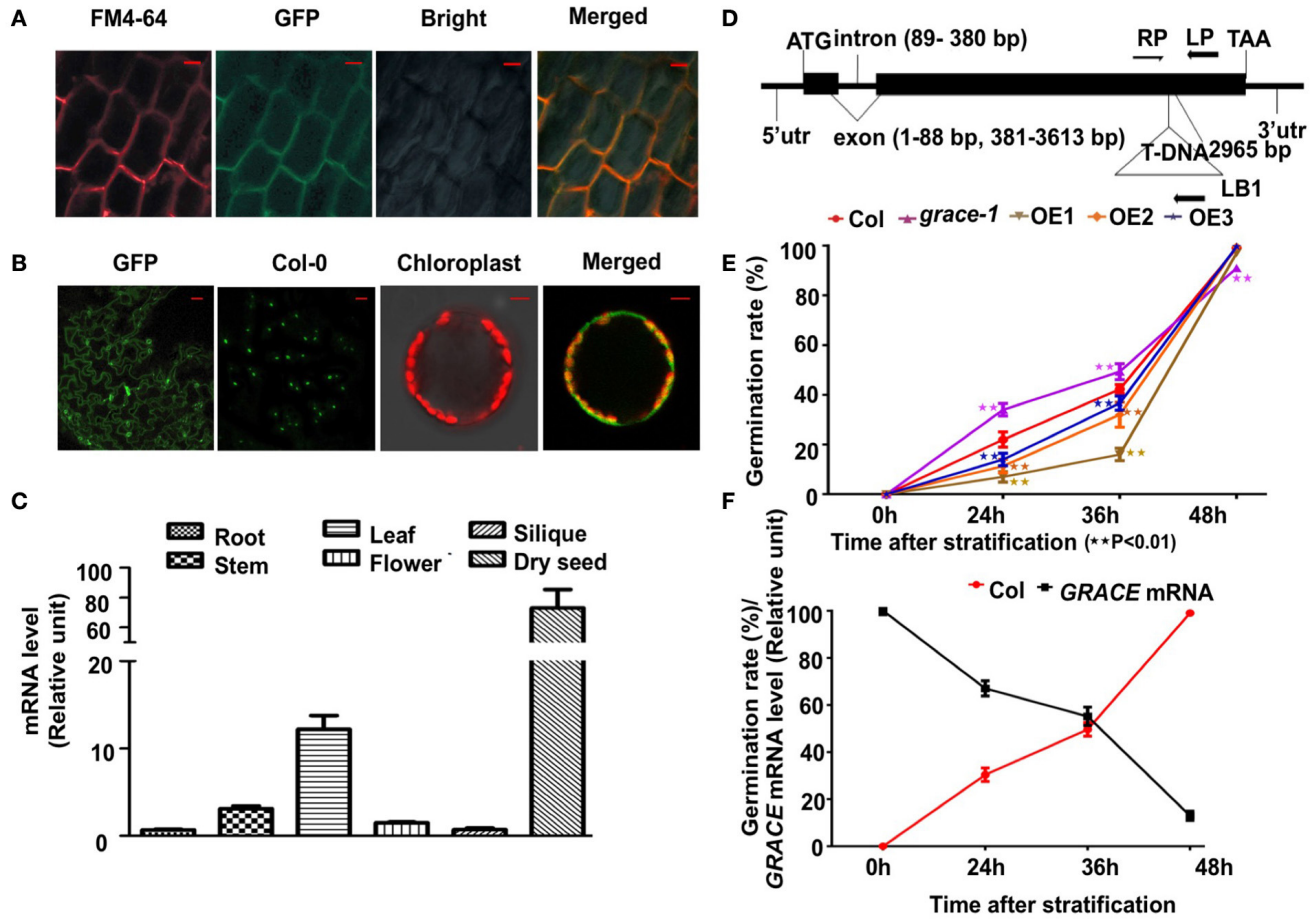
*GRACE* is mainly expressed in seeds and leaves and encodes a membrane protein. **(A)** Confocal microscopy images of the green fluorescent protein (GFP)-tagged GRACE in the root cells of 10-day-old overexpressing transgenic line OE1 seedlings. A portion of the GRACE-GFP and a plasma membrane marker FM4-64 are co-localized to the plasma membrane (Merged) with the bright field (Bright) (bars, 10 μm). **(B)** Confocal microscopy images of GRACE-GFP on membrane of epidermis and protoplasts of 2-week-old OE1 plants. GFP, GRACE-GFP on membrane of epidermis; Col-0, no GFP fluorescent for control (bars, 50 μm). The right two panels of images are false-colored to denote GFP and chlorophyll (red) fluorescence. Chloroplast, chlorophyll fluorescence (detected by DAPI detector) merged with protoplast bright field; Merged, GRACE-GFP fluorescence merged with chlorophyll fluorescence (bars, 5 μm). **(C)** Gene expression of *GRACE* is significantly higher in dry seed and rosette leaves than other tissues. Expression profiles of *GRACE* in various tissues were determined by real-time PCR. Plant materials include dry seeds (Dry seed), roots of 10-day-old plants (Root), stems/rosette leaves/flowers (Stem/Rosette/Flower) of 4-week-old plants and siliques (Silique) from 7-week-old *A. thaliana* wild type Col-0 seedlings. At least three times the experiments were repeated with the same results. **(D)** The T-DNA insertion site of *grace-1* (SAIL_865_E07, *A. thaliana* wild type Col-0 background). Lines and Boxes represent introns and exons, separately. LP, the left genomic primer; RP, and right genomic primer; LB1, left border primer for T-DNA. The T-DNA segment is near 2,965 bp of the open reading frame. bp, base pair. **(E)** Germination rates of different genotypes scored on 1/2 MS medium from 24 to 72 h after stratification in dark. Obvious emergence of radicals was counted as germination. Germination rates of Col-0, *grace-1* and OE1/2/3 are displayed in red/magenta/khaki/orange/violet, respectively. Each value is the mean ± SE of three determinations. Student's *t*-test was used to compare the germination rates of each genotype with those of the wild type Col-0 (^*^P < 0.05, ^**^P < 0.01). **(F)** Negative relativity between germination rates and *GRACE* transcript levels in Col-0 with respect to time after stratification, consistent with **(D)**. *GRACE* mRNA levels are displayed in black and germination rates are shown in red. Each value is the mean ± SE of three determinations.

We generated several *GRACE*-overexpressing lines with a C-terminal GFP under the control of an N-terminal constitutive CaMV-35S promoter and chose 3 of them, OE1/2/3 (gradient descent at transcript and protein levels) for analyses. The three transgenic lines were further confirmed by real-time PCR and western blot (Supplementary Figures 1A,B). To determine the subcellular localization of GRACE, 10-day-old OE1 seedlings were used for confocal microscopy analysis. As shown in Figure 1A, the 35S::GRACE-GFP fusion protein (from root cells) co-localized with the plasma membrane marker FM4-64. And in Figure 1B, GFP fluoresce could be observed on membrane of epidermis and protoplasts of OE1 leaves. These results suggest that *GRACE* encodes a membrane protein, as predicted by an LRR-RLK. Analysis of *GRACE* expression profiles showed that *GRACE* was expressed in the organs/tissues tested but significantly higher in dry seeds and rosette leaves than in other tissues (Figure 1C). These results are consistent with previous histochemical analysis of GUS activity and microarray data (Schmid et al., 2005; Wu et al., 2016).

### *GRACE* negatively regulates seed germination

We then isolated a T-DNA insertion knock-down mutant line (SAIL_865_E07; *A. thaliana* wild type Col-0 background) of the gene *At1g74360*, named *grace-1* (Figure 1D). The expression level of *GRACE* is greatly decreased in the knock-down mutant *grace-1* as compared to that in wild type Col-0 (Supplementary Figure 1A). To investigate the effect of knock-down of *GRACE* on seed germination, we planted the seeds of Col-0, *grace-1* and OE1/2/3 on 1/2 MS medium and examined their germination rates after 72 h-stratification in dark. As shown in Figure 1E, the seed germination rates of OE1/2/3 are considerably lower than that of Col-0. Conversely, the seed germination rate is strikingly promoted in *grace-1* (Figure 1E) as compared to that in Col-0. These results indicate that *GRACE* functions to delay seed germination and protect dormancy of *Arabidopsis*.

Given the high expression of *GRACE* in dry seeds (Figure 1C), we investigated whether expression of *GRACE* varies during germination. While abundant in dry seeds, the expression level of *GRACE* is substantially reduced after 24 h-stratification, and only ~10% of *GRACE* gene expression is detected after 48 h-stratification (Figure 1F, Supplementary Figure 1C). In further support of *GRACE*-mediated inhibition of seed germination, its reduced expressions are negatively correlated with the increased germination rates after stratification (Figure 1F). These results are consistent with our data from OE1/2/3 showing that increased GRACE protein expressions greatly reduce germination rates of *Arabidopsis* (Figure 1E).

### *GRACE* positively regulates cell expansion

**Figure 2:**
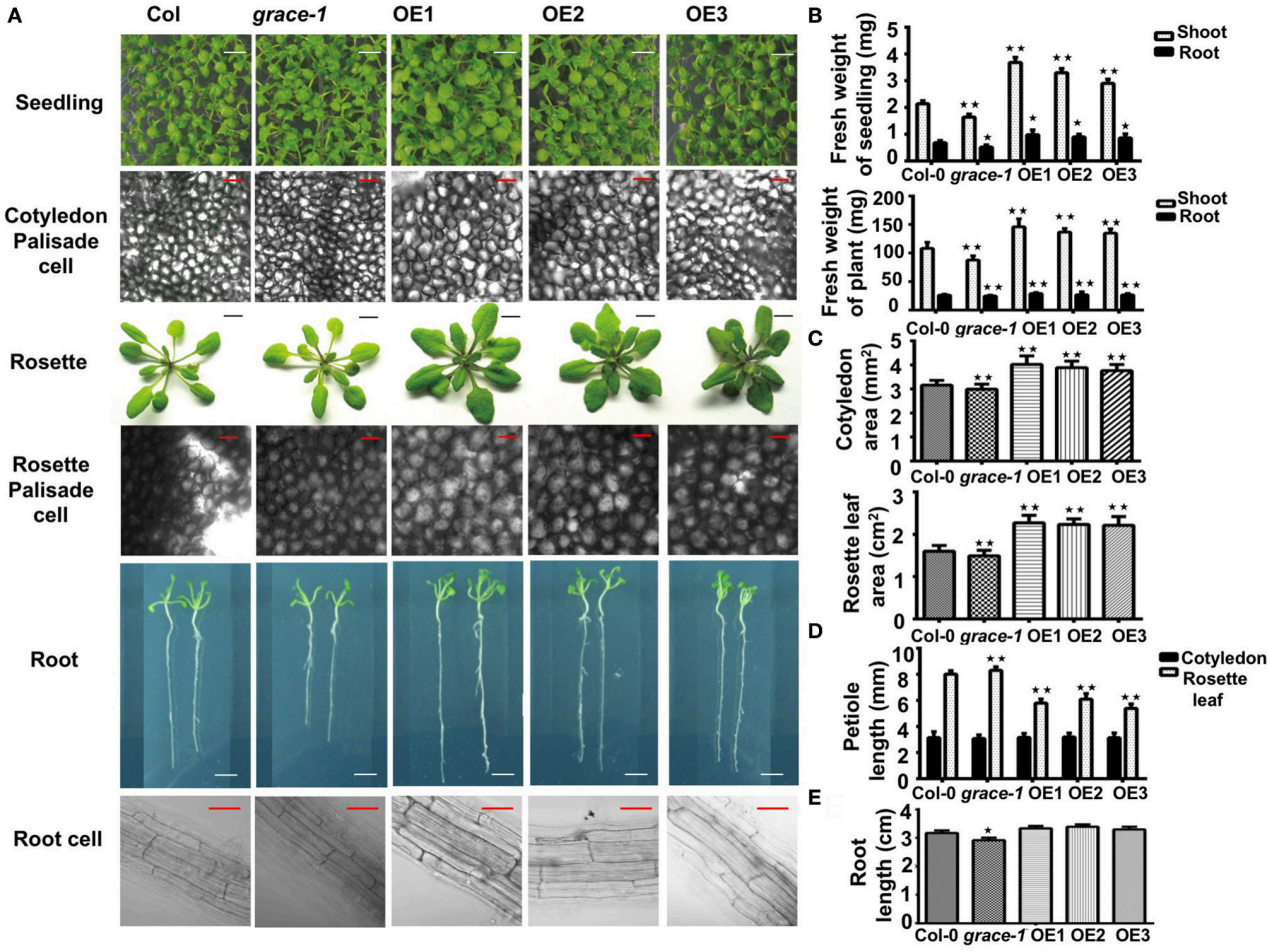
GRACE is involved in cell expansion and root development. **(A)** Images of seedlings, palisade cells, rosette leaves, root, and root cells. Seedling, early seedling growth of 10-day-old Col-0, *grace*-1 and OE1/2/3, which were directly planted on 1/2 MS medium (bars, 0.5 cm). Palisade cell, palisade cells of fully expanded cotyledons from 10-day-old seedlings and 4-week-old rosette leaves (bars, 50μm). Rosette, rosette leaves of 4-week-old seedlings (transferred to soil after 10-day growth on 1/2 MS medium) (bars, 1 cm). OE1/2/3 display visibly enlarged cotyledons, palisade cells, rosette leaves with stubbier petioles, while *grace-1* shows the reverses, in comparison with Col-0. Root (bars, 0.5 cm) and root cell (bars, 50 μm), from 10-day-old seedlings. **(B)** Statistic analysis of fresh weight of shoot/ root of 10-day-old seedlings and 4-week-old plants. **(C)** Statistic analysis of area of 10-day-old cotyledon and 4-week-old rosette leaf. Cotyledon, fully expanded cotyledon; rosette leaf, the 6th fully expanded rosette leaf. **(D)** Statistic analysis of petiole length of cotyledon/rosette leaf. **(E)** Root length of different genotypes on 1/2 MS medium without treatment. Each value is the mean ± SE of three determinations. Student's *t*-test was used to compare the germination rates of each genotype with those of the wild type Col-0 (^*^P < 0.05, ^**^P < 0.01).

To investigate whether different germination rates would be coupled with affected seedlings growth, we made morphological analyses of plants grown from the seeds of Col-0, *grace-1*, and OE1/2/3. As shown in Figure 2A, OE1/2/3 display visibly enlarged cotyledons, and rosette leaves with stubbier petioles. Statistical analyses of seedling/plant weight, cotyledon/rosette leaf area, petiole length and root length, support the phenotypes (Figures 2B–E). These results suggest a positive role played by *GRACE* in regulation of organ size and growth. In further support of this conclusion, the opposite phenotypes, though less striking, were observed in *grace-1*. The size differences of these organs exist from seedling to adult stage in different genotypes. Despite the size differences, morphologies of the cotyledon of *grace-1* and OE 1/2/3 remain similar to those of Col-0 (Figure 2A). It is of interest to note that, despite their delay in seed germination and enlarged organs, OE1/2/3 display no accelerated bolting, early blossoming and premature senescence or death (not shown), suggesting that the larger leaves in OE1/2/3 result from promoting cell growth rather than affecting life cycle of *Arabidopsis*. To further support this conclusion, we morphologically and cytologically analyzed 10-day-old seedlings and 4-week-old plants of different genotypes using microscope scanning. The data from these assays show that OE 1/2/3 have appreciably larger palisade cells than Col-0. By contrast, those cells from *grace-1* plants are comparatively smaller, though less striking, than those of Col-0 in *Arabidopsis* (Figure 2A).

Roots of 10-day-old seedlings grown on 1/2 MS medium were measured for statistical analysis. As expected, *grace-1* shows visibly shorter roots compared with Col-0. However, there is no significant difference between OE1/2/3 and Col-0 at the level of *P* < 0.05, Student's *t*-test (Figure 2E). Lower transcript levels in roots compared to those in rosette leaves might suggest that *GRACE* has more important role in leave growth than in root elongation (Figure 1C). Moreover, in our observation of root cell, we found that cell size does contribute to the root length, other than thickness (Figure 2A). Admittedly, current data cannot tell whether other factors such as cell proliferation have impact on the phenotype, more experiments will be executed in the future.

### ABA/JA/BL have crosstalk with *GRACE*-mediated signaling

**Figure 3:**
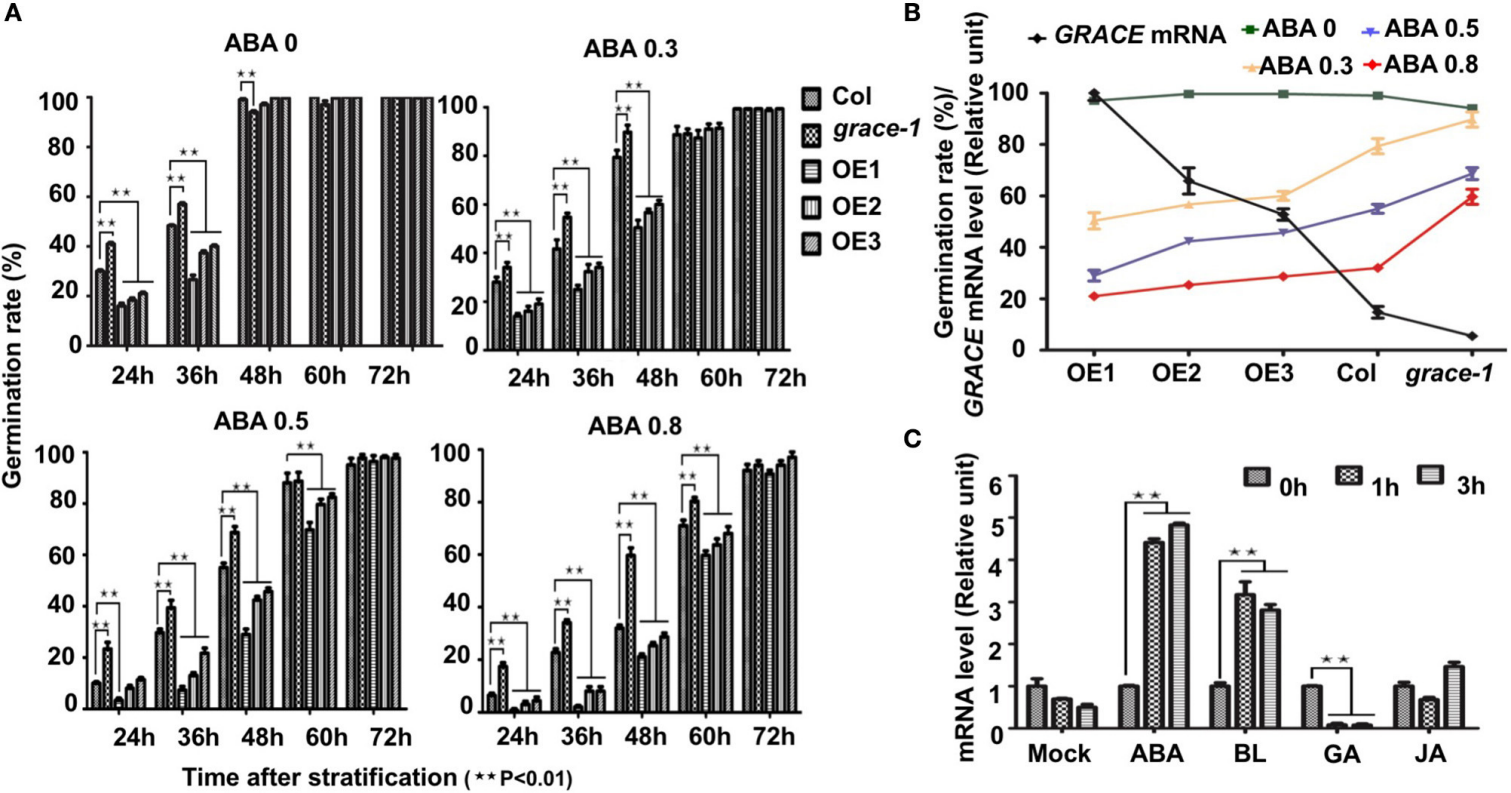
Germination rates of different genotypes with ABA treatment. **(A)** Germination rates of different genotypes scored on 1/2 MS medium containing 0/0.3/0.5/0.8 μM (±) ABA from 24 to 72 h after stratification in dark. Each value is the mean ± SE of three determinations. Student's *t*-test was used to compare the germination rates of each genotype with those of the wild type Col-0 (^*^P < 0.05, ^**^P < 0.01). **(B)** Negative correlation of *GRACE* mRNA levels and germination rates of different genotypes (Col-0, *grace-1*, OE1/2/3) with a series of *GRACE* transcript levels at 48 h after stratification. Col-0, *grace-1* and OE1/2/3 were dispersed on 1/2 MS medium containing 0/0.3/0.5/0.8 μM (±) ABA. Each value is the mean ± SE of three determinations. **(C)** Analysis of *GRACE* expression levels after different treatments. 10-day-old Col-0 seedlings grown on 1/2 MS were sprayed with100 mM (±) ABA/5 μM BL /100 μM GA/1.5 mM JA solutions or mock solution (0 μM, as a control), and sampled 0/1/3/5/7 h after the spraying for analysis. The expression levels of *GRACE* enhance after ABA/BL treatments while decreased under GA treatment. All experiments were repeated at least three times. Student's *t*-test was used to compare the statistical significance of the treated samples with those of mock (^**^P < 0.01).

To investigate whether common phytohormones participate in *GRACE*-mediated germination and cell expansion, we examined effects of them on germination rates in Col-0, *grace-1*, and OE1/2/3. Abscisic acid (ABA) inhibits the germination rates of *Arabidopsis* (Finch-Savage and Leubner-Metzger, 2006). Our genetic data show that treatment of OE1/2/3 with exogenous ABA results in further reduction in their germination rates. By contrast, ABA-inhibited germination is partially rescued in *grace-1* (Figures 3A,B). Given a negative role of *GRACE* in regulating germination, these results may be not surprising. However, germination inhibition by ABA and *GRACE*-overexpression does not seem to result from their mere additive effects, because expression of *GRACE* could be significantly promoted in the ABA-treated plants as compared to that of the non-treated plants (Figure 3C), indicating that *GRACE* expression positively responds to ABA signaling.

**Figure 4:**
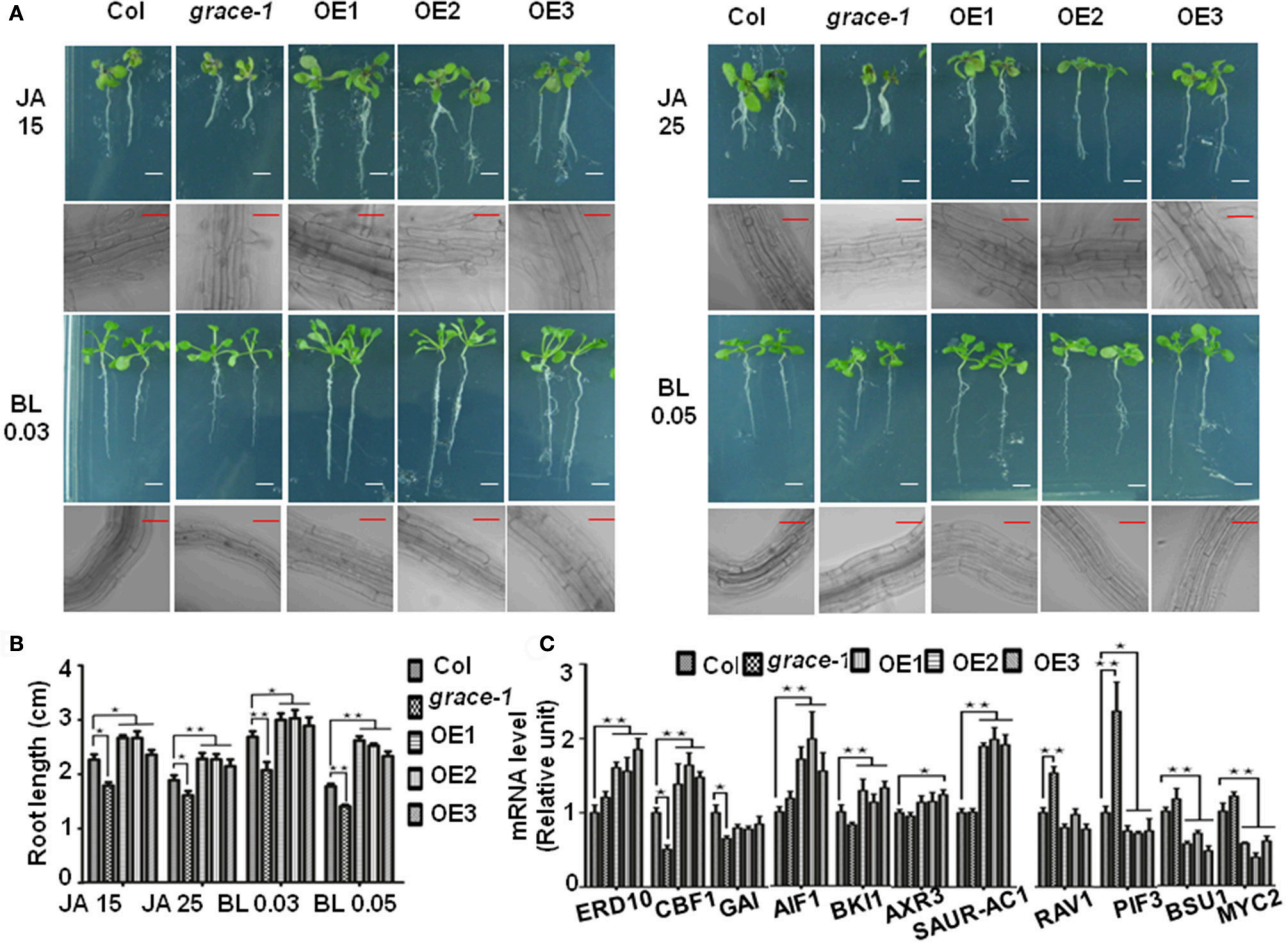
Root lengths of different genotypes with JA/BL treatments and altered *GRACE* expression levels influence the transcript levels of a subset of growth-contributing genes. **(A)** Root length and root cell of 10-day-old seedlings subjected to 15/25 μM JA and 0.03/0.05 μM BL application (bars, 0.5 cm for root, 50 μm for root cell). The sensitivity of root length responding to JA/BL is reduced along with the enhancing of *GRACE* transcript levels, which resulted in root length of Col-0 and *grace-1* decreasing much more than OE1/2/3. **(B)** Statistic analysis of root length of 10-day-old seedlings on 1/2 MS medium supplemented to 15/25 μM JA or 0.03/0.05 μM BL application. Each value is the mean ± SE of three biological experiments. Student's *t*-test was used to compare the root lengths of each genotype with those of the wild type Col-0 (^*^P < 0.05, ^**^P < 0.01). **(C)** Altered *GRACE* expression levels influence the transcript levels of a subset of growth-contributing genes. Gene expression in the 10-day-old Col-0, *grace*-1 and OE1/2/3 planted on 1/2 MS medium were sampled for analysis and detected by real-time PCR. Each value is the mean ± SE of three determinations. Student's *t*-test was performed to test statistical significance of means to compare the transcript levels of different genes in each genotype with those of the wild type Col-0 (^*^P < 0.05, ^**^P < 0.01).

Besides, jasmonate (JA) (Vilhar et al., 1991; Staswick et al., 1992) and brassinolide (BL) (Kim et al., 2007) would inhibit roots elongation of *Arabidopsis* in sufficient concentrations. In our observation, exogenously applied JA or BL made the root elongation of Col-0 and *grace-1* significantly decrease much more than that of OE1/2/3 (Figures 4A,B). In other word, the inhibition of root length responding to exogenous JA/BL could be significantly reduced by enhancing *GRACE* transcript levels. Treatment of BL can induce *GRACE* mRNA and jasmonate (JA) had little effect on the expression of *GRACE* in different genotypes at *P* < 0.05 level (Figure 3C).

Further supporting previous observations, altered *GRACE* expression levels affect a subset of transcript levels of growth-contributing genes (Figure 4C, Supplementary Figure 1D). The transcript levels of detected genes in Col-0, *grace-1*, and OE1/2/3 were determined by real-time PCR (primers in Supplementary Table 1). The transcript levels of ABA positive responder *ERD10* (Kim and Nam, 2010), GA negative regulators *CBF1* (Siddiqua and Nassuth, 2011) and *GAI* (Boccaccini et al., 2014), BL negative regulators *AIF1* (Wang et al., 2009) and *BKI1* (Wang and Chory, 2006; Jiang et al., 2015), auxin (AUX) positive regulators/responders *AXR3* (Leyser et al., 1996; Rinaldi et al., 2012) and *SAUR-AC1* (Gil et al., 1994) were significantly up-regulated in OE1/2/3 or down-regulated in *grace-1* compared with Col-0 (Figure 4C). Moreover, ABA/BL negative regulators *RAV1* (Hu et al., 2004), GA positive regulator *PIF3* (Stewart et al., 2011; Bai et al., 2014), BL positive regulator *BSU1* (Ryu et al., 2010; Kim et al., 2016) and JA positive regulator *MYC2* (Abe et al., 2002; Dombrecht et al., 2007; Fernandez-Calvo et al., 2011; Vos et al., 2013) were essentially up-regulated in *grace-1* or down-regulated in OE1/2/3 compared with Col-0 (Figure 4C). The above results were generally consistent with the roles of GRACE playing in seed germination inhibition, plant growth regulation, and response to relative phytohormones.

Additionally, the transcript levels of ABA positive regulators *ABF2* (Uno et al., 2000; Yoshida et al., 2010) and *MYB2* (Abe et al., 2002; Guo and Gan, 2011; Yu et al., 2012), GA negative regulator *RGL3* (Wild et al., 2012; Shi et al., 2016), BL negative regulator *IBH1* (Nagatoshi et al., 2016) and JA negative regulator *JAZ1* (Goossens et al., 2015) were up-regulated in *grace-1* or down-regulated in OE1/2/3 compared with Col-0 (Supplementary Figure 1D). The transcript levels of BL positive regulators *BZR1* (Ryu et al., 2007) and *PRE1* (Mara et al., 2010) were up-regulated in OE1/2/3 and down-regulated in *grace-1* compared with Col-0 (Supplementary Figure 1D). Based on current data, *GRACE* negative responds to BL (root elongation inhibition). While, detection results of some BL downstream components are reminiscent of negative feedback and dose-dependent biphasic manner of BL, which is not contradictory with phenotype of endogenous signals. Clearly, many more studies are needed to understand the mechanisms underlying the regulations.

### Overall structure of extracellular domain of GRACE

Though some genetic evidence had been collected to illuminate the physiological functions of GRACE, we wanted to further find out molecular clues for GRACE through structural study.

**Figure 5:**
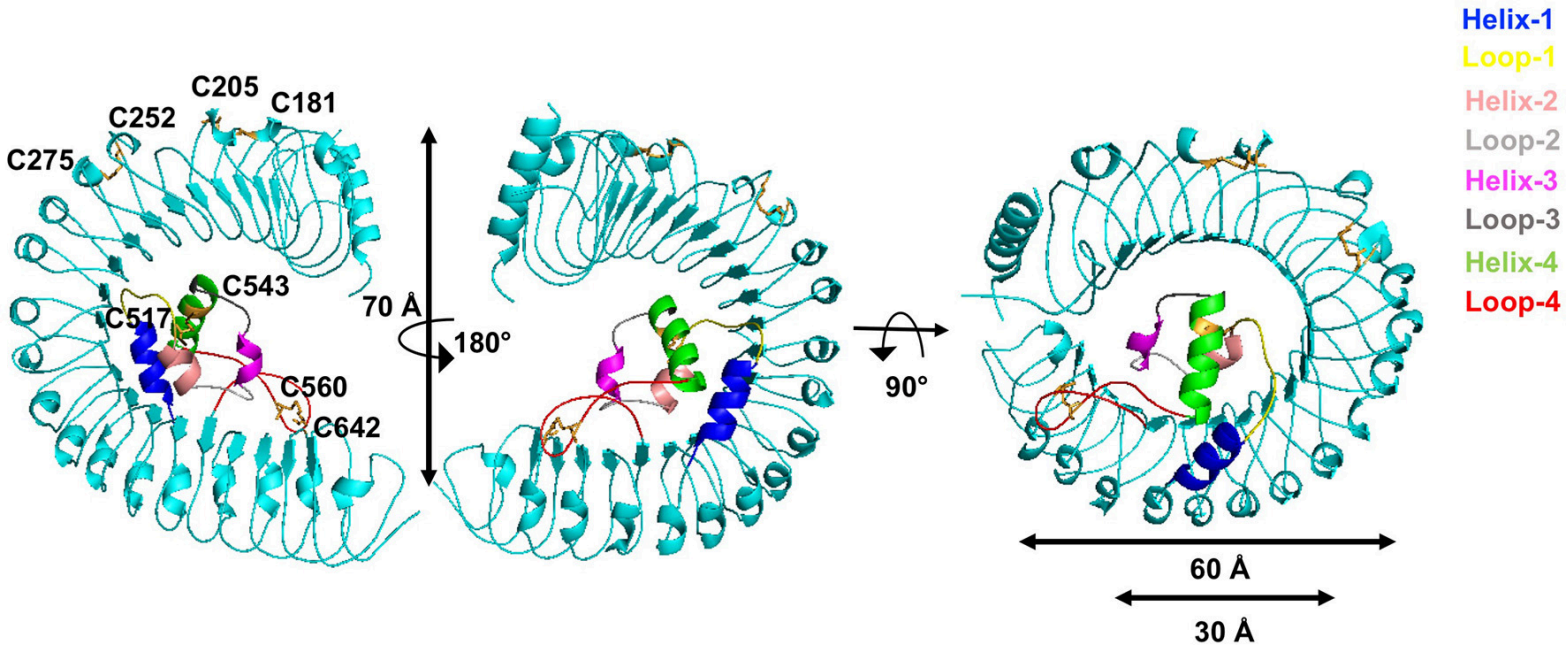
GRACE-LRR adopts a right-handed super-helical structure with an ID packing against the solenoid. Overall structures of GRACE-LRR in different orientations. Disulfide bonds are shown in stick and colored in orange. The LRR solenoid is shown in aquamarine. The Helix-1,−2,−3,−4 and Loop-1,−2,−3,−4 are shown in blue, salmon, magenta, green, yellow, gray, deep gray, and red, respectively. “N” and “C” indicate the N- and C-terminus, respectively.

We then determined the monomer crystal structure of the extracellular domain of *A. thaliana* GRACE (GRACE-LRR) (Supplementary Figures 2A,B) with a resolution of 3.0 Å (Supplementary Table 2). GRACE-LRR contains 22 LRRs, assembling into a twisted right-handed super-helical solenoid structure, completing a whole turn with outer diameter of ~60 Å and a rise of ~70 Å (Figure 5, Supplementary Figure 3). As predicted by sequence, residues 495–572 form an island domain (ID), which packs against the interior of the solenoid of GRACE-LRR via extensive interactions without disrupting the single solenoid (Figure 5, Supplementary Figure 4). Notably, a disulfide bond is formed between the ID and the solenoid structure, further stabilizing their interactions. The ID consists of three helices, with Helix-1 mainly interacting with a lateral side of the solenoid and Helix-2 and−3 binding to the inner surface (Figure 5). Structure-based sequence alignment shows that the residues involved in the interaction between the ID and the solenoid of GRACE-LRR highly conserved among GRACE homologs (Supplementary Figures 4, 5), suggesting their indispensable roles in protein folding.

### Structural comparison indicates GRACE function as a receptor recognizing a new potential signal

**Figure 6:**
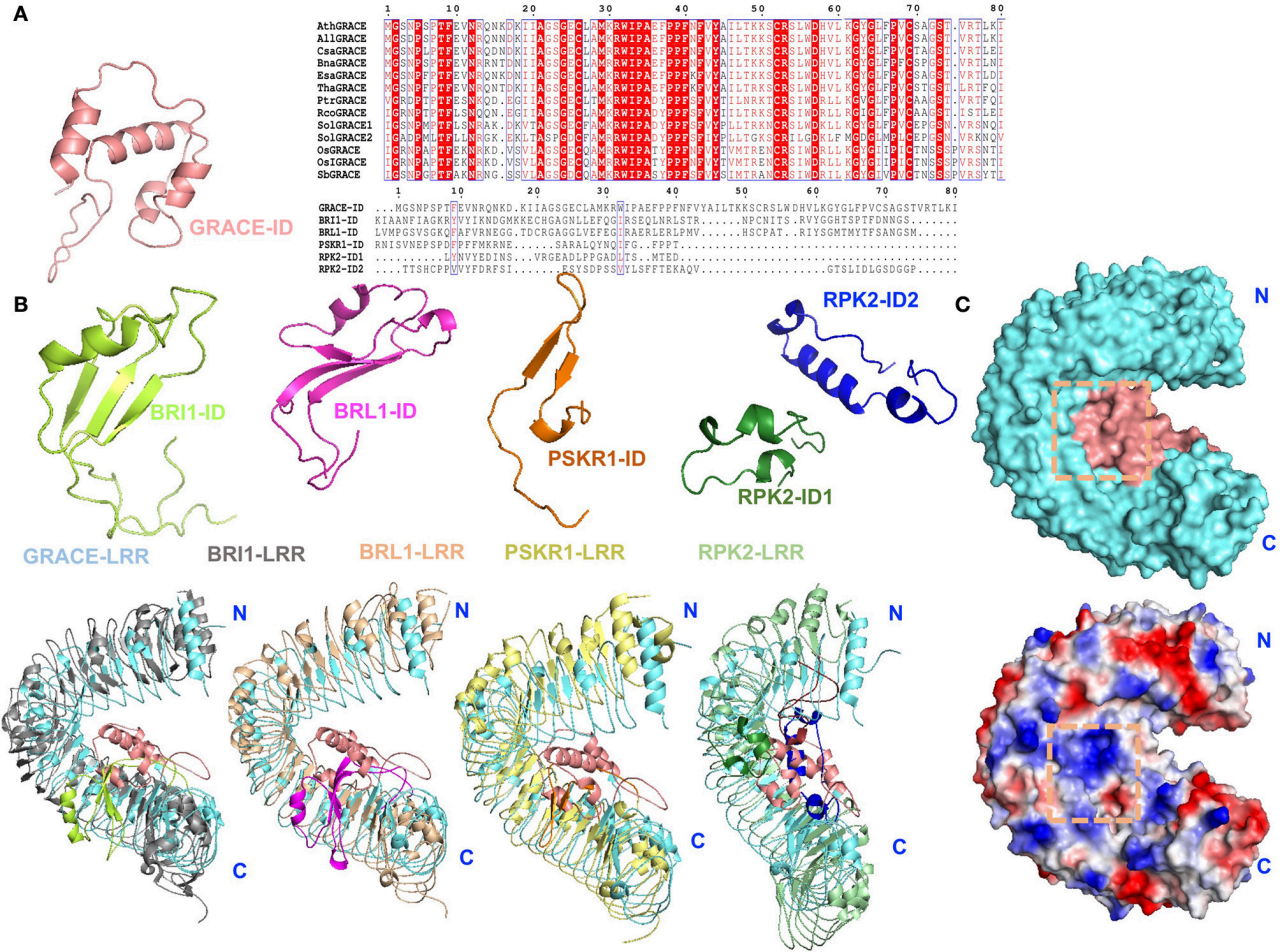
Structural comparison and surface representations of GRACE-LRR. **(A)** Alignments of GRACE-ID and its homologs from plants or other LRR-RLK-IDs in *Arabidopsis*. Left: structure of GRACE-ID. Middle: sequence alignment of GRACE-ID and its homologs from plants by ClustalX 2.1 and colored by Espript 3.0. Conserved and similar residues are boxed with red ground and red font, respectively. Ath, *Arabidopsis thaliana*; All, *Arabidopsis lyrata subsp*. Lyrata; *Csa, Camelina sativa*; Esa, *Eutrema salsugineum*; Bna, *Brassica napus*; Tha, *Tarenaya hassleriana*; Ptr, *Populus trichocarpa* (Western balsam poplar); Rco, *Ricinus communis* (Castor bean); Sol, *Solanum lycopersicum* (Tomato); Os, *Oryza sativa* (Japonica cultivar-group); OsI, *Oryza sativa subsp*. Indica; Sb, *Sorghum bicolor*. Right: sequence alignment of GRACE-ID and other LRR-RLK-IDs in *Arabidopsis*. **(B)** Structural comparison of GRACE-ID and -LRR with other reported LRR-RLK-IDs or extracellular domain of LRR-RLKs. The different folding of reported IDs. GRACE-ID, BRI1-ID, BRL1-ID, PSKR1-ID, RPK2-ID1, and RPK2-ID2 are shown in salmom, limon, magentas, orange, forest, and blue, respectively. The LRR solenoids of BRI1, BRL1, PSKR1, RPK2 are colored in gray, wheat, pale yellow, and pale green, respectively. **(C)** Surface representations of GRACE-LRR (shown in the same orientation of the left panel of Figure 4A). Top: potential ligand binding site formed by ID and the inner surface of LRR solenoid, highlighted in orange squares. The LRR solenoid and ID are shown in aquamarine and salmon, respectively. Bottom: surface electrical property of GRACE-LRR. White, blue and red indicate neutral, positive and negative surfaces, respectively. “N” and “C” indicate the N- and C-terminus, respectively.

Thus far, several structures of LRR-RLKs with an ID including BRI1 and PSKR1 and RPK2 have been solved. Given the previous studies, the ID appears to be an ideal candidate scaffold for ligand(s) binding of LRR-RLKs (Hothorn et al., 2011; She et al., 2011, 2013; Wang et al., 2015). Based on sequence alignment, GRACE-ID is highly conserved among homologs, but strikingly different from currently known LRR-RLK-IDs (She et al., 2011; Song et al., 2014; Wang et al., 2015; Figure 6A). Structural comparison of GRACE-LRR with these LRR-RLKs reveals that all these IDs mainly interact with the inner surfaces of the LRR solenoids near the C-terminal (Figure 6B). Despite the similarity, substantial differences exist among these IDs. The most striking one is the structural flexibility of the IDs in the absence of ligands. Structural studies indicate that the IDs in both BRI1 and PSKR1 are structurally flexible in their ligand-free forms (She et al., 2011; Wang et al., 2015). In particular, the ID of PSKR1 is completely disordered in the absence of its ligand PSK. Formation of the PSKR1-ID is induced by PSK binding (Wang et al., 2015). BR binding also has a role in stabilizing the ID of BRI1, leading to formation of a clear-cut BR binding pocket on the surface of BRI1 (Hothorn et al., 2011; She et al., 2011). In sharp contrast, the whole ID of GRACE-LRR is well-defined by electron density (Figure 6C). This likely results from the extensive interaction including one disulfide bond between the ID and the solenoid of GRACE-LRR (Figure 5). Thus, ligand binding sites, if exist, involving the GRACE-ID and GRACE-LRR are pre-formed.

In addiction, the surface created by the ligand(s)-free GRACE-ID spans about 16 LRRs (from LRR7 to LRR22; Figure 5), whereas the ligand binding BRI1/BRL1-ID and PSKR1-ID are located only five LRRs from the membrane surface (She et al., 2011, 2013; Wang et al., 2015). Compared to known extracellular domain of LRR-RLKs, the overall structure of GRACE-LRR has a similar size with those of BRI1-LRR (25 LRRs)/BRL1-LRR (24 LRRs) in diameter and rise, with fewer LRRs (22 LRRs; Figure 5B). It is likely due to the longer ID of GRACE than BRI1/BRL1-ID and that only one disulfide bond is formed between two consecutive repeats of GRACE-LRR affecting tightness of the solenoid (Figure 5), when five pairs in BRI1-LRR/BRL1-LRR (Hothorn et al., 2011; She et al., 2011, 2013).

## Discussion and perspectives

Establishment of seed germination is critical for plant life cycle. Phytohormones have been shown to play important roles in regulating these processes. However, the molecular mechanisms underlying remain less well-defined. In the current study, we performed genetic and structural studies to characterize the functionally unknown LRR-RLK GRACE from *Arabidopsis*, which belongs to LRR X (Shiu and Bleecker, 2001a,b; Gou et al., 2010; Supplementary Figure 6A). Our genetic data support a role played by *GRACE* in inhibiting seed germination probably through maintaining their dormancy. On the other hand, we also show that *GRACE* plays a role in promoting cell expansion. Consistently, analysis of *GRACE* expression profiles show that *GRACE* is mainly expressed in dry seeds and rosette leaves. Down regulation of *GRACE* expression promotes seed germination; accordantly, its overexpression results in delayed seed germination. Evidence for a positive role of *GRACE* in cell expansion comes from our observation of plant cotyledon and leaf morphology and palisade cell. Whether and how these two different functions of GRACE are associated with each other remain unknown. One possible explanation is that GRACE regulates downstream signaling by a dose-dependent biphasic manner: high doses of upstream signal resulting from high expression of GRACE in seeds inhibits germination and low doses of that in rosette leaves promotes growth. The seeming contradictory roles that GRACE plays in different periods during plants growing are complementary to each other in a certain sense, for thorough mature contributing to post-germination growth, and germination inhibition of premature embryos insuring seed quality.

We also provide evidence showing that expression of *GRACE* is greatly reduced with the progression of seed germination. The negative correlation between *GRACE* expression and seed germination raises the possibility that down-regulation of *GRACE* expression is a mechanism to relieve GRACE-mediated inhibition during seed germination. For LRR-RKs are rarely reported mediating signaling by degradation receptor itself, so it possibly presents a novel regulation mechanism of LRR-RLKs in germination. This is reminiscent of ABA-mediated inhibition of seed germination, in which endogenous ABA gradually increases during seed dormancy and gradually decreases during germination, and the expression of *GRACE* is significantly promoted by ABA (Figure 3C). Conversely, gibberellin (GA) accumulates to break dormancy (Yamauchi et al., 2004; Finch-Savage and Leubner-Metzger, 2006) and the expression of *GRACE* is substantially reduced by GA (Figure 3C). The content of endogenous ABA and GA would result in strengthening the role of GRACE. We treated plants with Paclobatrazol (PAC, a GA synthesis inhibitor), but the germination rates of OE1/2/3 exceed those of the wild type Col-0 in 48 h and those of *grace-1* are considerably inhibited compared to Col-0 (Supplementary Figures 6B,C). It is likely that the signal mediated by *GRACE* is inhibited by PAC or negative feedback would be activated under GA synthesis defects, while it needs further evidences.

Our data show that GRACE protein is mainly membrane localized, consistent with the prediction that it functions as an RLK. The available data indicate LRR-RLKs generally act as receptors to perceive internal or external ligands to activate their kinase activity for cellular signaling. In this respect, however, it remains completely unknown whether GRACE, like other known LRR-RLKs, recognizes a ligand to initiate signaling for regulation of physiological processes. Structural analysis shows that the ID of GRACE-LRR is better defined as compared to other IDs of LRR-RLKs. Although the identities of GRACE ligands remain unknown, this structural observation suggests that potential ligand binding sites around the ID of GRACE are pre-formed. The topological characters presented by the surface of this cavity, such as the electropositive, hydrophilic nature, and restricted size, imply that it might sense a small molecular as ligand rather than peptides/nucleic acids/lipids, to induce subsequent processes. Alternatively, it also remains possible that GRACE senses a signal that can make modifications on it without traditional continuous binding, thus allowing GRACE to homo- or hetero-oligomerize with its co-receptors for its activation as shown in other LRR-RLKs. As exemplified by PSKR-1-mediated signaling, ligand is not necessarily directly involved in oligomerization of an LRR-RLK for its activation. SERKs have been demonstrated to act as co-receptors with many LRR-RLKs including BRI1 and PSKR1, two members of LRR X subfamily (Santiago et al., 2013; Sun et al., 2013; Han et al., 2014; Wang et al., 2015). It is coincidental to note that SERKs contain five LRRs and the IDs of BRI1/PSKR1 are inserted into the last four LRRs in the C-terminal. Similarly, GRACE-ID locates at the last five LRRs, it will be interesting to investigate whether SERK members play roles in *GRACE*-mediated pathway. Given the visible functions and expression specificity of GRACE, the potential signal appears to be broadly available in various tissues, especially seeds and leaves. Considering the high homology of the ID among different species, the potential signal of GRACE is expected to be conserved in plants.

Our functional studies fill the blank of the functionally unknown, ID-containing LRR-RLKs and the structural information of GRACE-LRR shows a unique ID structure, which broaden our understanding toward LRR-RLKs and provide insights into GRACE-mediated signaling. Although, current information renders it difficult to associate the structure of GRACE with its functions in depth, our functional and structural evidences are together provided for indicating the potential expression specificity and topological characters of the unknown ligand(s). Further investigations are required to identify the ligand(s) of GRACE and elucidate the molecular basis of initiation for GRACE-mediating transduction pathway, which might reveal a novel regulating pathway and can be used for more efficient agricultural practice, such as designing of growth regulators for leaf vegetables and improving methods of seed conservation, in the future.

## Materials and methods

### Plant materials and growth conditions

*A. thaliana* wild type Col-0 (control) and the T-DNA insertion line *grace-1* (SAIL_865_E07) in Col-0 background were obtained from Arabidopsis Biological Resource Center (ABRC). Mutant *grace-1* was genotyped by PCR (primers in Supplementary Table 1). Seeds were surface sterilized in 4% NaClO_3_ for 25 min followed by more than 5 times of wash using sterile H_2_O. The disinfected seeds were dispersed on 1/2 MS (Murashige & Skoog) media (Sigma) containing 1% (w/v) agar, 3% (w/v) sucrose, pH 5.8–6.0, stratificated in dark for 3 days at 4°C and transferred to a growth chamber under 80 μmol photons m^−2^ s^−1^ (12 h-light/12 h-dark) for 10 days followed by transplanting in compost soil under 120 μmol photons m^−2^ s^−1^ (16 h-light/8 h-dark), cool white fluorescent lamps, at 22°C, 60% relative humidity. Information and phenotypes of another T-DNA insertion line *grace-2* (SAIL_859_H01, Col-0 background, from ABRC, primers in Supplementary Table 1) are supplemented in Supplementary Figures 7, 8.

### Generation of overexpression constructs and plant transformation

For stable transgenic plants overexpressing *GRACE*, the constract was generated by inserting the open reading frame (ORF) of *GRACE* (residues 1–1,106) into the pCAMBIA-1300-221 vector, which harbors a N-terminal constitutive Cauliflower Mosaic Virus (CaMV) 35S promoter and a C-terminal green fluorescent protein (GFP)-encoding sequence (primers in Supplementary Table 1, Col-0 cDNA used as template). The construct was verified by sequencing and transformed into 5-week-old *A. thaliana* wild type Col-0 by the floral infiltration method (Clough and Bent, 1998) using the construct via GV3101 strain of *Agrobacterium tumefaciens*. The transgenic lines were isolated by hygromycin. The homozygous T3 seeds overexpressing *GRACE* were used for further analysis.

### Analysis of protein subcellular localization and gene expression profiles

For subcellular localization analysis, epidermis and protoplasts of 2-week-old OE1 plants were generated, and roots of 10-day-old OE1 seedlings were immersed in 5 μM FM4-64 for 10 s, and then they were observed for GFP fluorescence and photographed by confocal microscope Zeiss LSM780 (Olympus). Ten-day-old seedlings were used as plant materials for determination of *GRACE* transcript levels in *grace-1*, Col-0, and *GRACE*-overexpressing transgenic lines. The total proteins were extracted from the 10-day-old seedlings and immunoblotting was performed with anti-GFP serum. For analysis of *GRACE* expression profiles in different periods of germination, about 0.1 ml dry seeds were used for single sample. The seeds were sampled 0/24/36/48 h after 72 h-stratification in dark. Col-0 seedlings (10-day-old) grown on 1/2 MS medium were sprayed with different solutions [100 mM (±) ABA/150 mM NaCl/5 μM BL/100 μM GA/1.5 mM JA] or mock solution (0 μM, as a control), and sampled 0/1/3/5/7 h after the spraying for analysis.

### Phenotypic analysis

Col-0, *grace-1*, and OE1/2/3 were used as plant materials for phenotypic analysis. For seed germination assay, about 100 seeds were dispersed on 1/2 MS medium supplemented with 0/0.3/0.5/0.8 μM (±) ABA. Germination (obvious emergence of radicals) was recorded at the indicated times after 72 h-stratification in dark. For seedling growth observation, about 50 seeds were planted on 1/2 MS medium and transferred to soil after 10 days. The palisade cells at the central region of fully expanded cotyledons (about 10 days after stratification) were observed as representative tissues to determine cell size. Sections of the palisade cells and root cells were photographed by confocal microscope Zeiss LSM780 (Olympus). For root length measurement, about 30 seedlings of 10-day-old grown on 1/2 MS medium with 0/15/25 μM JA, 0/0.03/0.05 μM BL were measured, respectively. The length from the junction of hypocotyl and root to the root tip was counted. For petiole length, cotyledon/rosette leaf area, seedling/plant shoot/root fresh weight measurement (weighing immediately detached), about 30 seedlings of 10-day-old grown on 1/2 MS medium or 4-week-old plants (the 6th fully expanded rosette leaf, as shown in Supplementary Figures 9A,B) in soil were measured. Software Image J (National Institutes of Health, http://rsb.info.nih.gov/ij) was used for measurements of root/petiole length, and cotyledon/leaf area. Electronic analytical balance was used for weight measurements. All measurements were repeated at least three times. Student's *t*-test was used to show statistical differences.

### Quantitative real-time PCR analysis

Ten-day-old seedlings were used as plant materials for analysis on downstream genes of *GRACE*. Total RNA of grinded plant materials in liquid nitrogen was extracted using a Total RNA Rapid Extraction Kit (BioTeke) and General Total RNA Extraction Kit (for seeds), incubated with RNase-free DNase I (NEB) at 37°C for 30 min to degrade genomic DNA and further cleaned by an RNA Purification Kit (BioTeke). Subsequently, 1 μg RNA was subjected to first-strand cDNA synthesis with Roche cDNA Synthesis Kit. Analysis was performed using the Real-Time System CFX96TM C1000 Thermal Cycler (BioRad) with SYBR Premix ExTaq (TaKaRa) by a DNA Engine Opticon 2-step thermal cycler in 10 μL volume. ACTIN2/8 genes were amplified as an internal control.

### Protein expression and purification

The construct of GRACE-LRR (residues 35–729) with an engineered N-terminal hemolin signal peptide and a fused C-terminal 6 × His tag was generated by standard PCR-based cloning strategy into the pFastBac™-1 vector (Invitrogen; primers in Supplementary Table 1, Col-0 cDNA used as template), and confirmed by sequencing. The secreted protein was expressed in High Five insect cells at 22°C using the Bac-to-Bac baculovirus expression system (Invitrogen). One liter of cells (1.8 × 10^6^ cells mL^−1^ cultured in the medium from Expression Systems) was infected with 20 mL baculovirus and harvested the supernatant from the media by centrifugation (4°C, 4,000 rpm, 15 min) after 60 h. The supernatant was purified using Ni-NTA column (Novagen) followed by gel filtration chromatography (Hiload 200, GE Healthcare) at 4°C in buffer containing 10 mM Bis-Tris pH 6.0 and 100 mM NaCl. Homogeneous samples from relevant fractions were applied to SDS-PAGE and visualized by Coomassie blue staining. For improving the diffraction ability of the crystals, the purified protein was digested with endoglycosidase F1 and F3 in 50 mM Na_3_PO_4_ at 18°C overnight, cleaned by gel filtration and further concentrated to about 10.0 mg·mL^−1^ for crystallization.

### Crystallization, data collection, structural determination, and refinement

Deglycosylated GRACE-LRR were generated by mixing equal volumes (1.0 μL) of protein and reservoir solution using the hanging-drop vapor-diffusion method. Diffraction quality crystals were obtained under the conditions of 0.2 M C_4_H_12_N_2_O_6_, 20% (w/v) Polyethylene glycol (PEG) 3,350 within 10 days at 18°C. For data collection, crystals were equilibrated in a cryoprotectant buffer containing reservoir buffer plus 10% (v/v) glycerol. All the diffraction data sets were collected on beam line BL17U1 using a CCD detector at the Shanghai Synchrotron Radiation Facility (SSRF). The data were integrated and scaled with HKL2000 (Otwinowski and Minor, 1997). Subsequently, the structure of the deglycosylated GRACE-LRR was solved by Molecular Replacement (MR) with PHASER included in CCP4 (Computational Project Number 4, 1994) using the structure of BRI1-LRR (PDB code: 3RGZ) as the initial search model. The model from MR was built with COOT (Emsley and Cowtan, 2004) and subjected to refinement by PHENIX (Adams et al., 2002). Finally, the model was refined to a resolution of 3.0 Å with Rwork = 22.6% and Rfree = 28.9%. All the structure figures were prepared using PYMOL (DeLano, 2002).

## Accession numbers

The *Arabidopsis* Information Resource (TAIR) accession number for *GRACE* is *At1G74360*, and the GenBank accession numbers for *GRACE* is NM_106096.4.

## Author contributions

JC, ZH, ZW, SL, and WS: Designed the experiments. ZW, SL, and WS: Performed the experiments. JC, ZH, ZW, SL, and WS: Analyzed the data; GL, HZ: Collected the X-ray data; JC: Solved the structure; SL, ZW: Edited the figures; JC, ZH, ZW, and WS: Wrote the manuscript.

### Conflict of interest statement

The authors declare that the research was conducted in the absence of any commercial or financial relationships that could be construed as a potential conflict of interest.

## Supplementary material

Supplementary Figure 1 **(A)** Real-time PCR analysis of *GRACE* transcript levels in the 10-day-old wild type Col-0, knock-down mutant *grace-1*, and *GRACE*-overexpression transgenic lines OE1/2/3. Each value is the mean ± SE of three biological determinations. **(B)** Western blotting of 10-day-old OE1/2/3 seedlings using anti-GFP. **(C)** Expression levels of *GRACE* are abundant in seeds under dry condition and during stratification. The experiments were repeated at least 3 times with the same results. **(D)** Supplementary data of altered *GRACE* expression levels influence the transcript levels of a subset of growth-contributing genes. Gene expression in the 10-day-old Col-0, *grace*-1 and OE1/2/3 planted on 1/2 MS medium were sampled for analysis and detected by real-time PCR. Each value is the mean ± SE of three determinations. Student's *t*-test was performed to test statistical significance of means to compare the transcript levels of different genes in each genotype with those of the wild type Col-0 (^*^*P* < 0.05, ^**^*P* < 0.01).

Supplementary Figure 2 The GRACE protein exists as monomers in solutions and crystals. **(A)** The GRACE protein exists as monomers in solutions. Top: schematic representation of GRACE. SP, signal peptide; NT, N-terminal cap; LRR, leucine rich repeat; ID, island domain; TM, transmembrane domain; KD, kinase domain. Left panel of the bottom: The gel filtration chromatogram of GRACE-LRR. The vertical and horizontal axes represent ultraviolet absorbance (λ = 280 nm) and elution volume (mL), respectively. The elution volume of RPK2-LRR is about 79 mL, corresponding to a molecular weight of ~100 kD. Right panel of the bottom: Coomassie blue staining of the peak fraction for SDS-PAGE. MM, molecular weight marker. **(B)** The GRACE protein exists as monomers in crystals. Dimers formed by two crystallographic symmetry-related monomers of GRACE-LRR. Two monomers are colored in magenta and aquamarine and their N- and C-terminus are indicated.

Supplementary Figure 3 Sequence alignment of regular LRRs in GRACE. The boundary of each LRR and its numbering are shown on the left side. The conserved residues are shown with yellow background. Cysteines forming disulfide bonds between LRRs are highlighted with magenta. The residues from the plant-specific motif GxL/i/vP (x stands for any amino acid; Di Matteo et al., 2003) in the LRRs are highlighted with black solid squares at the bottom.

Supplementary Figure 4 Detailed interactions between the ID and the LRR solenoid. **(A,B)** Detailed interactions of hydrogen bonds formed by main train atoms of the Loop-2 and −3 of the ID with those from the interface of the solenoid. Red dashed lines represent hydrogen bonds. The residues from the solenoid are shown in aquamarine and those from the ID are shown as indicated in Figure 5. **(C)** The hydrophobic residues from the solenoid uphold the hydrophobic cores of the ID from underneath. **(D)** The center of the hydrogen bonds network inside the ID. Red dashed lines represent hydrogen bonds. **(E)** Detailed hydrophobic core formed by the ID and the solenoid.

Supplementary Figure 5 Sequence alignment of extracellular domain of GRACE and its homologs from plants. Conserved and similar residues are boxed with red ground and red font, respectively. Cysteines forming disulfide bonds are highlighted with solid squares of same color at the bottom.

Supplementary Figure 6 **(A)**Phylogenetic clade of LRR X and VII subfamilies ran by MEGA 5.0. The members in light blue brackets are previous members of LRR X subfamily. **(B)** Germination rates of different genotypes scored on 1/2 MS medium containing 0/1/3/5 μM PAC from 24 to 72 h after stratification. Each value is the mean ± SE of three biological determinations. Student's *t*-test was used to compare the germination rates of each genotype with those of the ecotype Col-0 (^*^*P* < 0.05, ^**^*P* < 0.01). **(C)** Positive correlation of *GRACE* mRNA levels and germination rates of different genotypes at 60 h after stratification. Arabidopsis ecotype Col-0, *grace-1* and OE1/2/3 were dispersed on 1/2 MS medium containing 0/1/3/5 μM PAC. Each value is the mean ± SE of three biological determinations.

Supplementary Figure 7 Growth and germination phenotypes of *grace-2*. **(A)** Images of 10-day-old seedlings (bars, 0.5 cm), palisade cells of 10-day-old seedlings and 4-week-old plants (bars, 50 μm), 6-week-old rosette leaves (bars, 1 cm). Col-0 displayed larger cotyledons, palisade cells, rosette leaves, through less striking than that of *grace-1*. **(B)** The T-DNA insertion site of *grace-2* (SAIL_859_H01, *A. thaliana* wild type Col-0 background). The T-DNA segment is near 1,240 bp of the open reading frame. **(C)** Statistic analysis of fresh weight of shoot/ root of 10-day-old seedlings and 4-week-old plants. **(D)** Statistic analysis of area of 10-day-old cotyledon and 4-week-old rosette leaf. **(E)** Statistic analysis of petiole length of cotyledon/rosette leaf. **(F)** Germination rates of Col-0 and *grace-2* without treatment. **(G)** Germination rates of Col-0 and *grace-2* under ABA or PAC treatments. Each value is the mean ± SE of three determinations. Student's *t*-test was used to compare the germination rates of each genotype with those of the wild type Col-0 (^*^*P* < 0.05, ^**^*P* < 0.01).

Supplementary Figure 8 Root and root cell of 10-day-old seedlings. **(A)** Root and root cell of 10-day-old Col-0 and *grace-2* without treatment (root, bars, 0.5 cm; root cell, bars, 50 μm). **(B)** Root and root cell of 10-day-old Col-0 and *grace-2* under JA or BL treatment (μM). **(C)** Statistic analysis of root length of 10-day-old Col-0 and *grace-2*. **(D)** Some tested genes were altered in *grace-2*, compared to Col-0. Each value is the mean ± SE of three determinations. Student's *t*-test was used to compare the germination rates of each genotype with those of the wild type Col-0 (^*^*P* < 0.05, ^**^*P* < 0.01).

Supplementary Figure 9 Cotyledon and rosette leaf of different genotypes. **(A)** Cotyledons of 10-day-old seedling (bars, 0.5 cm). **(B)** Rosette leaf of 4-week-old plant (bars, 1 cm).
