# Supplementary figures and images for: Functional and Structural Characterization of a Receptor-Like Kinase Involved in Germination and Cell Expansion in Arabidopsis
Source: Front Plant Sci. 2017 Nov 22;8:1999. doi: 10.3389/fpls.2017.01999 (PMC5702872; doi:10.3389/fpls.2017.01999)

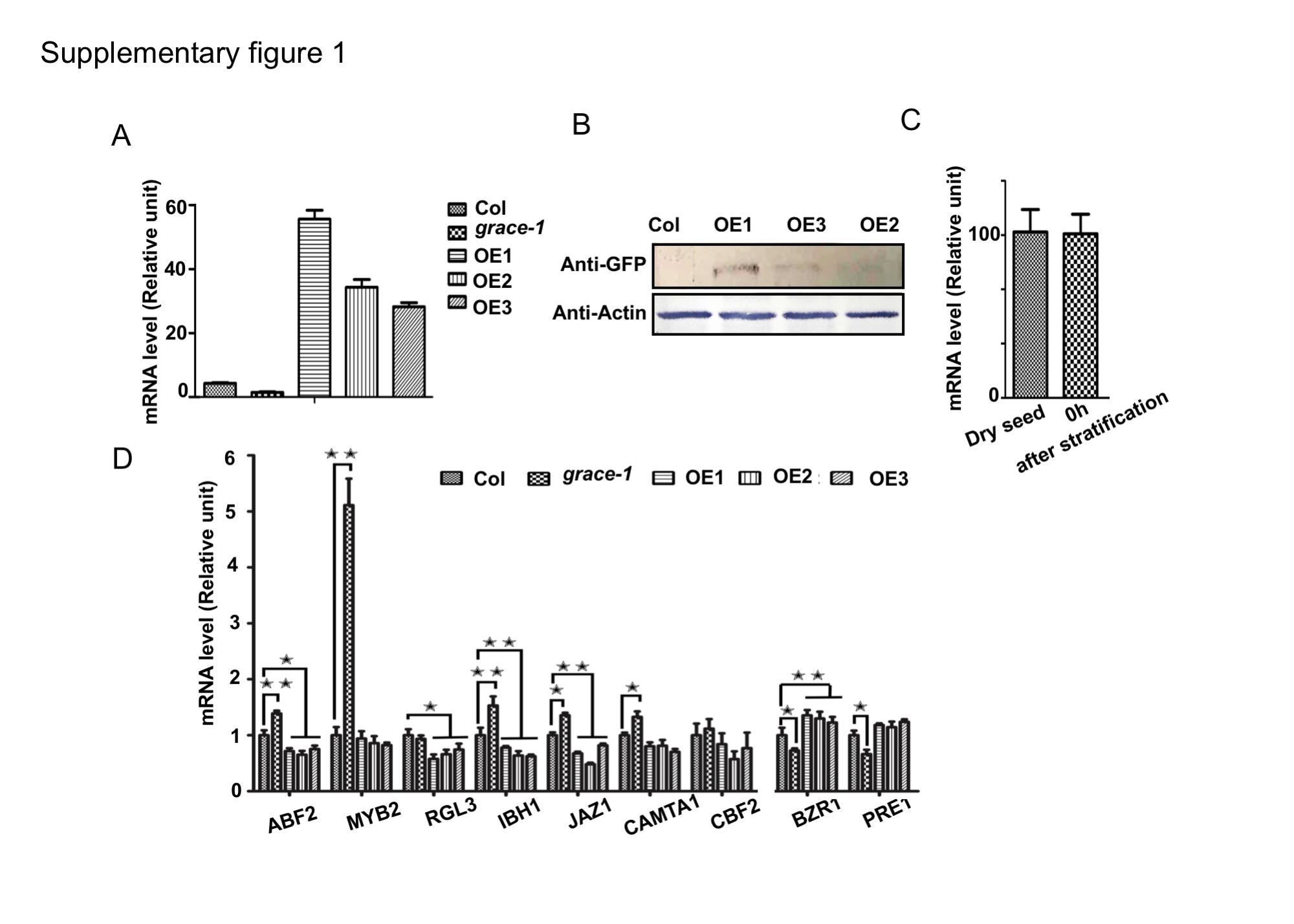

Supplement: Supplementary Figure 1 — (A) Real-time PCR analysis of GRACE transcript levels in the 10-day-old wild type Col-0, knock-down mutant grace-1, and GRACE-overexpression transgenic lines OE1/2/3. Each value is the mean ± SE of three biological determinations. (B) Western blotting of 10-day-old OE1/2/3 seedlings using anti-GFP. (C) Expression levels of GRACE are abundant in seeds under dry condition and during stratification. The experiments were repeated at least 3 times with the same results. (D) Supplementary data of altered GRACE expression levels influence the transcript levels of a subset of growth-contributing genes. Gene expression in the 10-day-old Col-0, grace-1 and OE1/2/3 planted on 1/2 MS medium were sampled for analysis and detected by real-time PCR. Each value is the mean ± SE of three determinations. Student's t-test was performed to test statistical significance of means to compare the transcript levels of different genes in each genotype with those of the wild type Col-0 (*P < 0.05, **P < 0.01). [file Image1.JPEG]

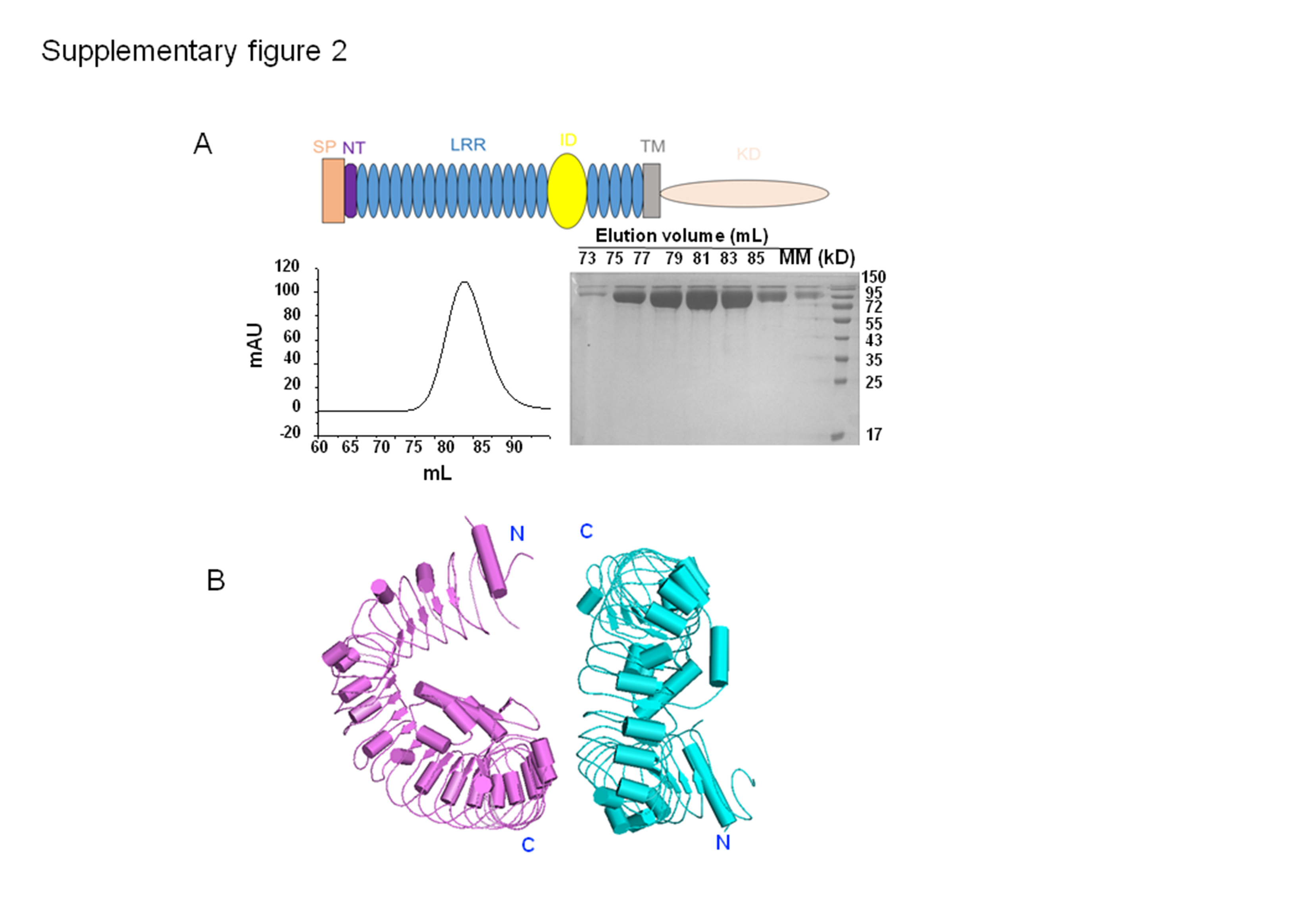

Supplement: Supplementary Figure 2 — The GRACE protein exists as monomers in solutions and crystals. (A) The GRACE protein exists as monomers in solutions. Top: schematic representation of GRACE. SP, signal peptide; NT, N-terminal cap; LRR, leucine rich repeat; ID, island domain; TM, transmembrane domain; KD, kinase domain. Left panel of the bottom: The gel filtration chromatogram of GRACE-LRR. The vertical and horizontal axes represent ultraviolet absorbance (λ = 280 nm) and elution volume (mL), respectively. The elution volume of RPK2-LRR is about 79 mL, corresponding to a molecular weight of ~100 kD. Right panel of the bottom: Coomassie blue staining of the peak fraction for SDS-PAGE. MM, molecular weight marker. (B) The GRACE protein exists as monomers in crystals. Dimers formed by two crystallographic symmetry-related monomers of GRACE-LRR. Two monomers are colored in magenta and aquamarine and their N- and C-terminus are indicated. [file Image2.TIF]

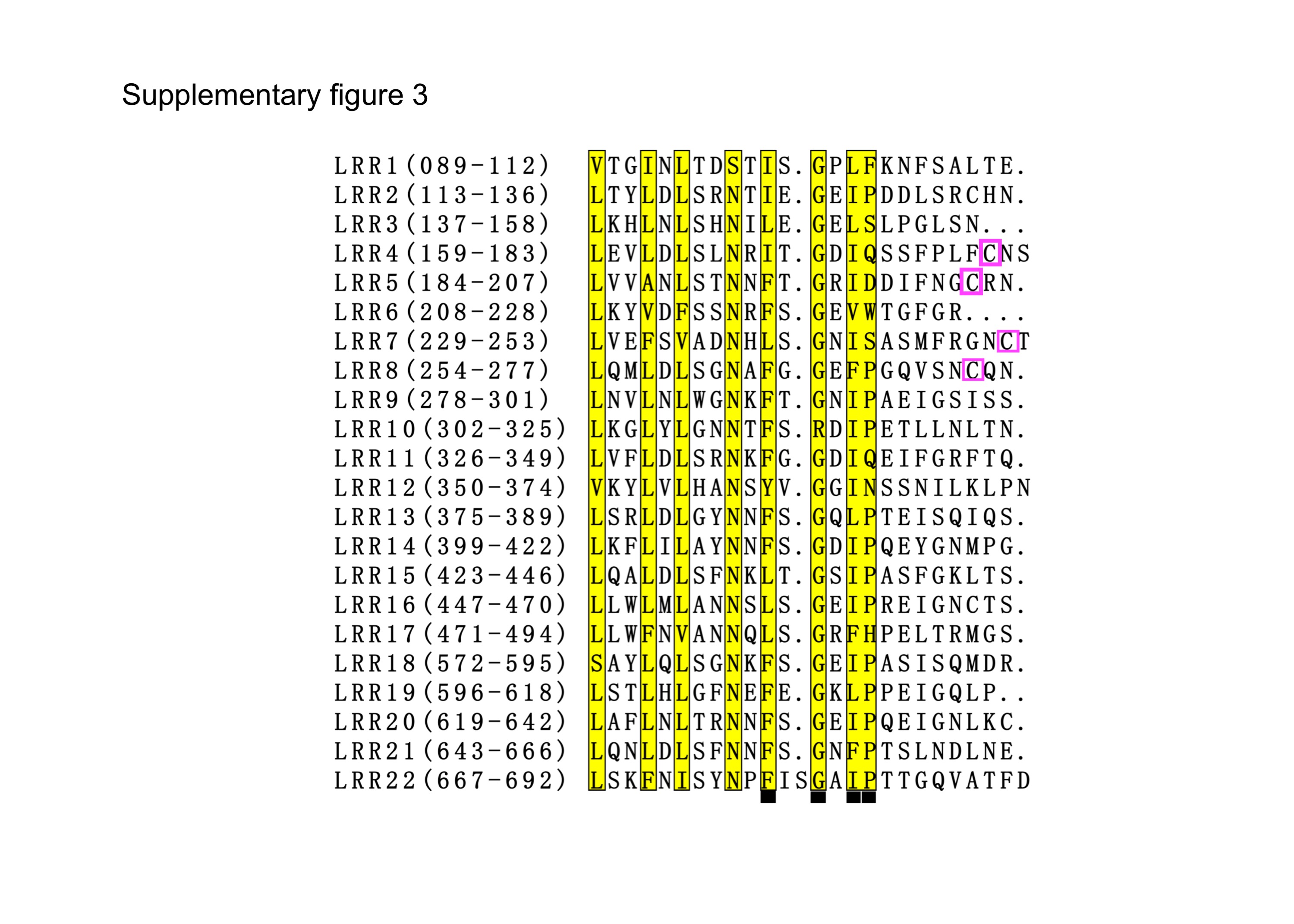

Supplement: Supplementary Figure 3 — Sequence alignment of regular LRRs in GRACE. The boundary of each LRR and its numbering are shown on the left side. The conserved residues are shown with yellow background. Cysteines forming disulfide bonds between LRRs are highlighted with magenta. The residues from the plant-specific motif GxL/i/vP (x stands for any amino acid; Di Matteo et al., 2003) in the LRRs are highlighted with black solid squares at the bottom. [file Image3.JPEG]

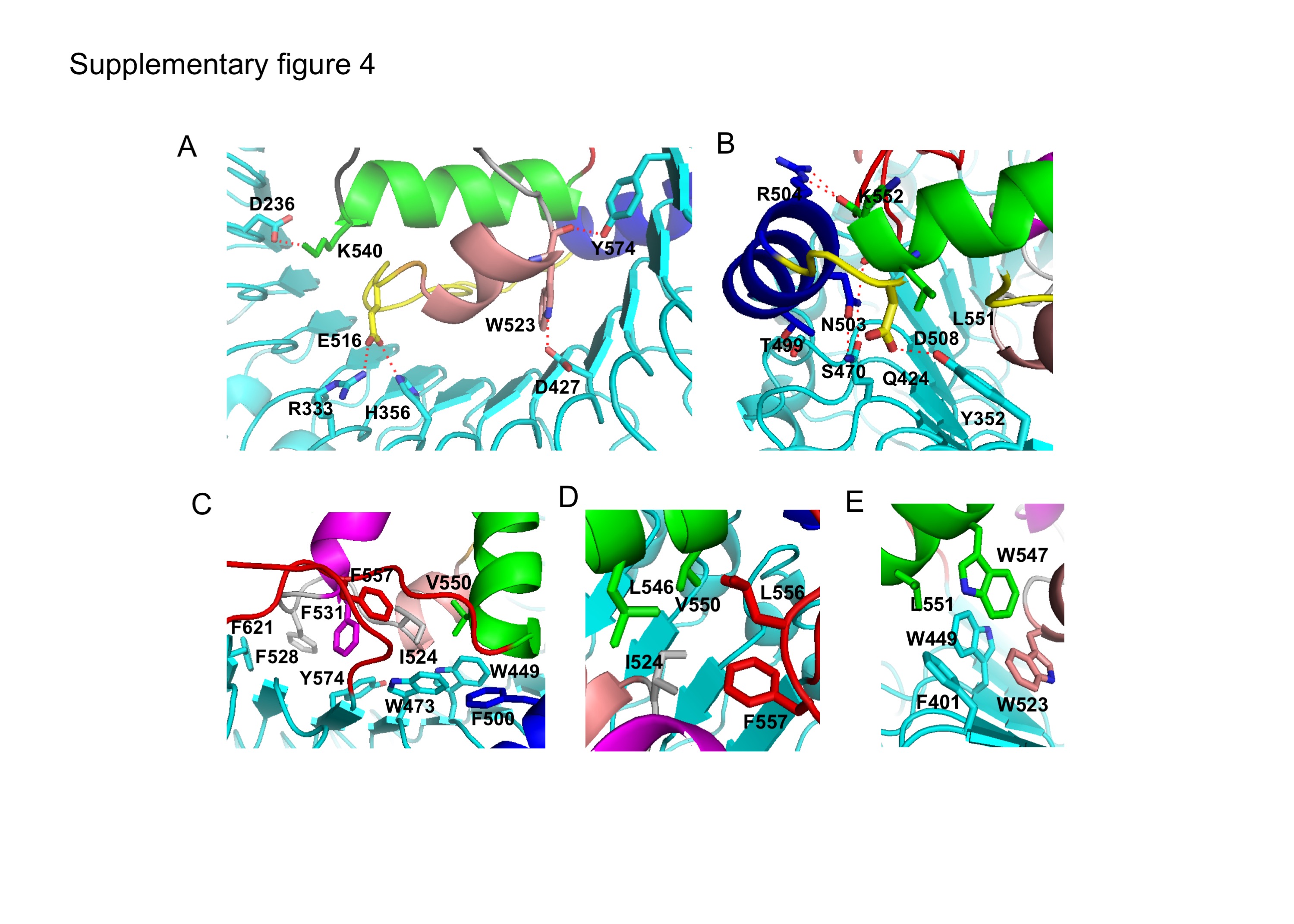

Supplement: Supplementary Figure 4 — Detailed interactions between the ID and the LRR solenoid. (A,B) Detailed interactions of hydrogen bonds formed by main train atoms of the Loop-2 and −3 of the ID with those from the interface of the solenoid. Red dashed lines represent hydrogen bonds. The residues from the solenoid are shown in aquamarine and those from the ID are shown as indicated in Figure 5. (C) The hydrophobic residues from the solenoid uphold the hydrophobic cores of the ID from underneath. (D) The center of the hydrogen bonds network inside the ID. Red dashed lines represent hydrogen bonds. (E) Detailed hydrophobic core formed by the ID and the solenoid. [file Image4.JPEG]

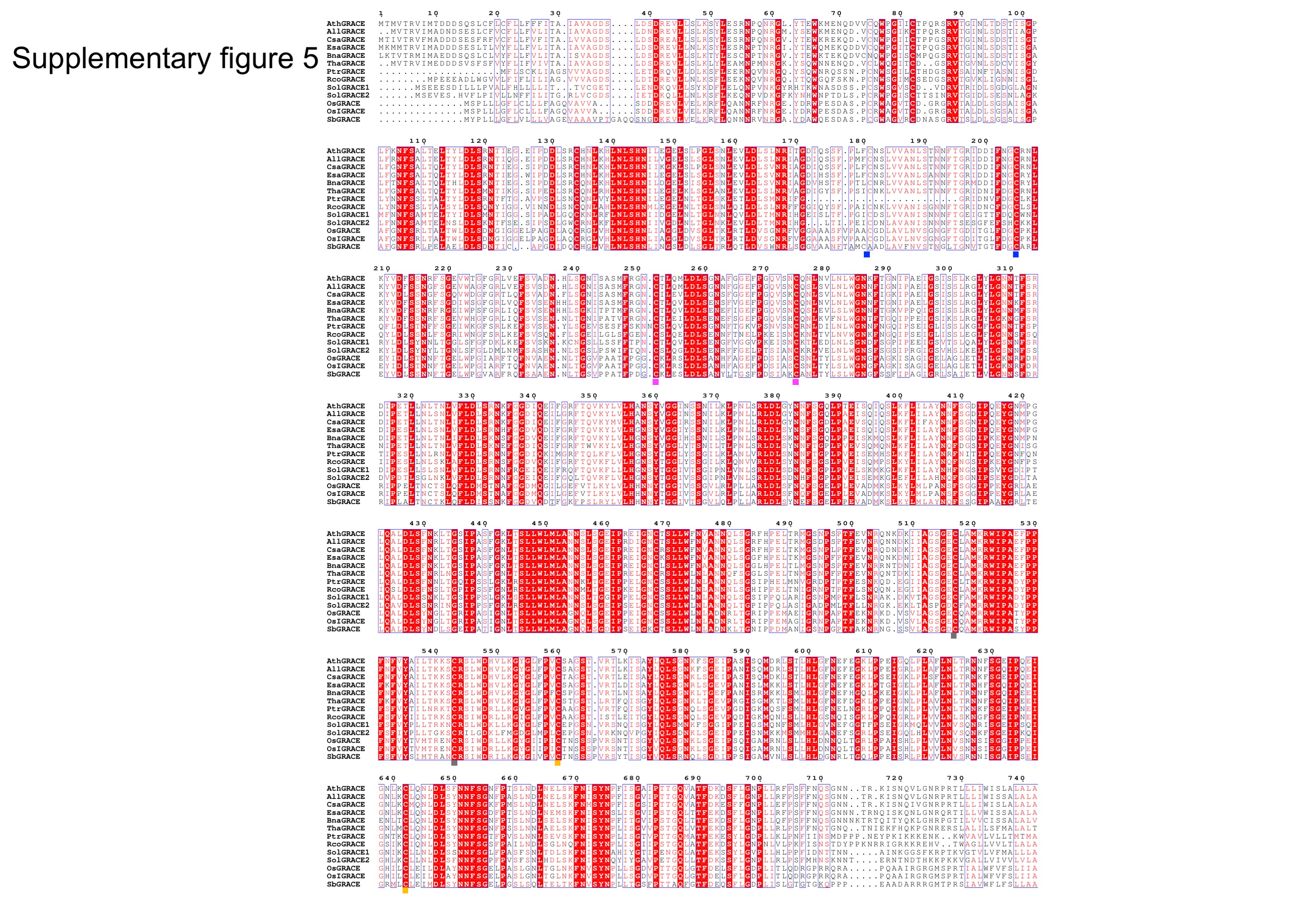

Supplement: Supplementary Figure 5 — Sequence alignment of extracellular domain of GRACE and its homologs from plants. Conserved and similar residues are boxed with red ground and red font, respectively. Cysteines forming disulfide bonds are highlighted with solid squares of same color at the bottom. [file Image5.JPEG]

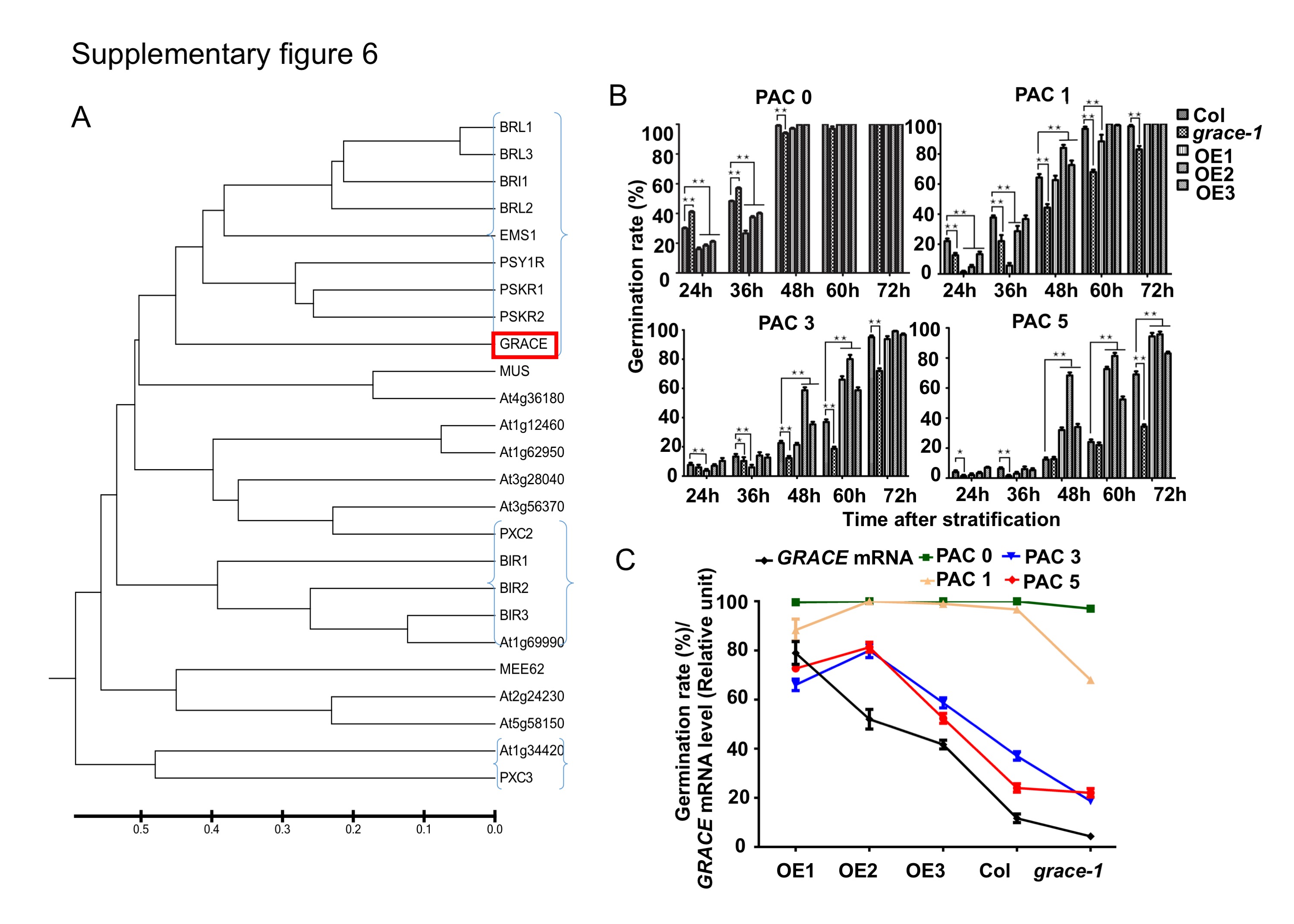

Supplement: Supplementary Figure 6 — (A)Phylogenetic clade of LRR X and VII subfamilies ran by MEGA 5.0. The members in light blue brackets are previous members of LRR X subfamily. (B) Germination rates of different genotypes scored on 1/2 MS medium containing 0/1/3/5 μM PAC from 24 to 72 h after stratification. Each value is the mean ± SE of three biological determinations. Student's t-test was used to compare the germination rates of each genotype with those of the ecotype Col-0 (*P < 0.05, **P < 0.01). (C) Positive correlation of GRACE mRNA levels and germination rates of different genotypes at 60 h after stratification. Arabidopsis ecotype Col-0, grace-1 and OE1/2/3 were dispersed on 1/2 MS medium containing 0/1/3/5 μM PAC. Each value is the mean ± SE of three biological determinations. [file Image6.JPEG]

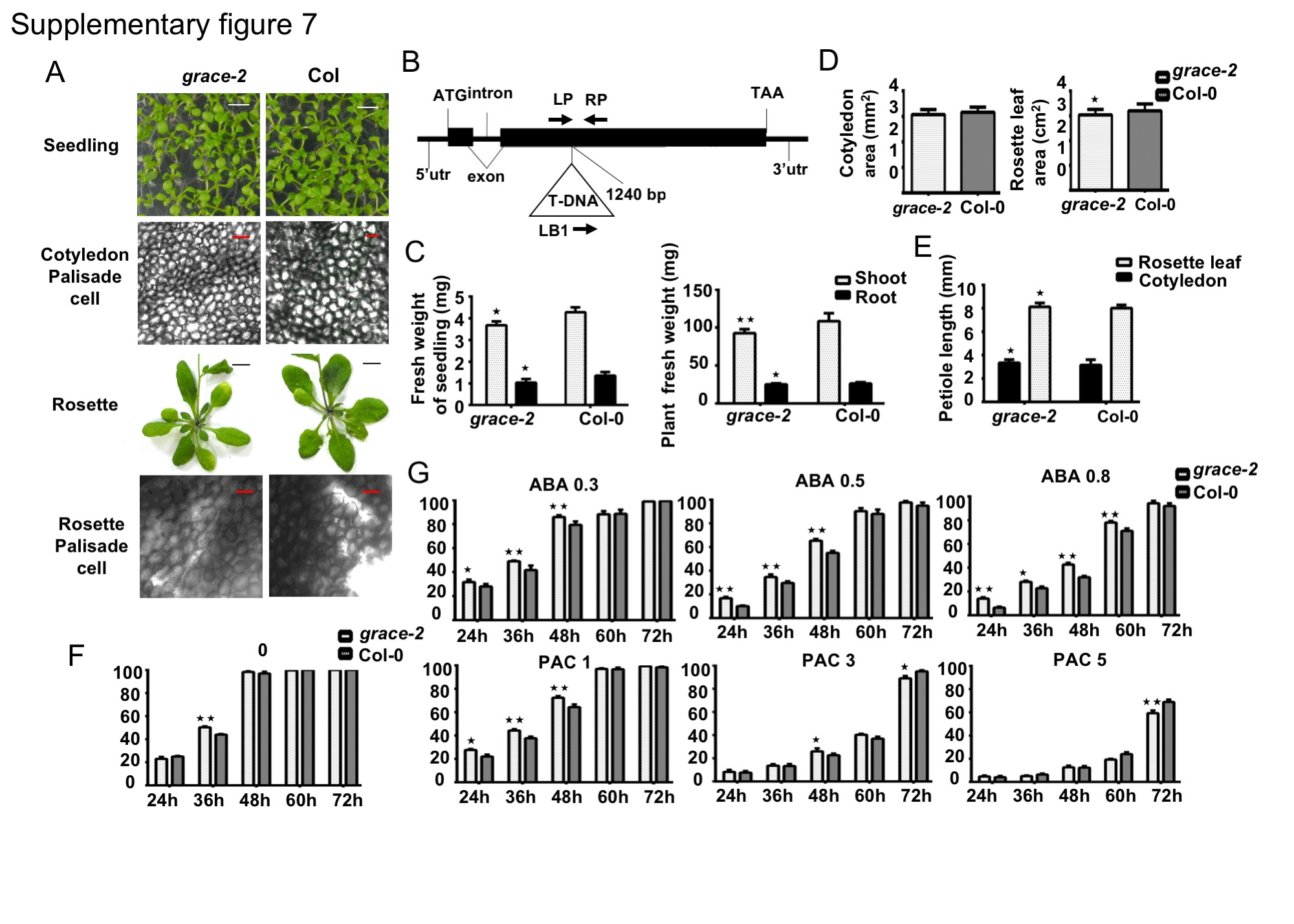

Supplement: Supplementary Figure 7 — Growth and germination phenotypes of grace-2. (A) Images of 10-day-old seedlings (bars, 0.5 cm), palisade cells of 10-day-old seedlings and 4-week-old plants (bars, 50 μm), 6-week-old rosette leaves (bars, 1 cm). Col-0 displayed larger cotyledons, palisade cells, rosette leaves, through less striking than that of grace-1. (B) The T-DNA insertion site of grace-2 (SAIL_859_H01, A. thaliana wild type Col-0 background). The T-DNA segment is near 1,240 bp of the open reading frame. (C) Statistic analysis of fresh weight of shoot/ root of 10-day-old seedlings and 4-week-old plants. (D) Statistic analysis of area of 10-day-old cotyledon and 4-week-old rosette leaf. (E) Statistic analysis of petiole length of cotyledon/rosette leaf. (F) Germination rates of Col-0 and grace-2 without treatment. (G) Germination rates of Col-0 and grace-2 under ABA or PAC treatments. Each value is the mean ± SE of three determinations. Student's t-test was used to compare the germination rates of each genotype with those of the wild type Col-0 (*P < 0.05, **P < 0.01). [file Image7.JPEG]

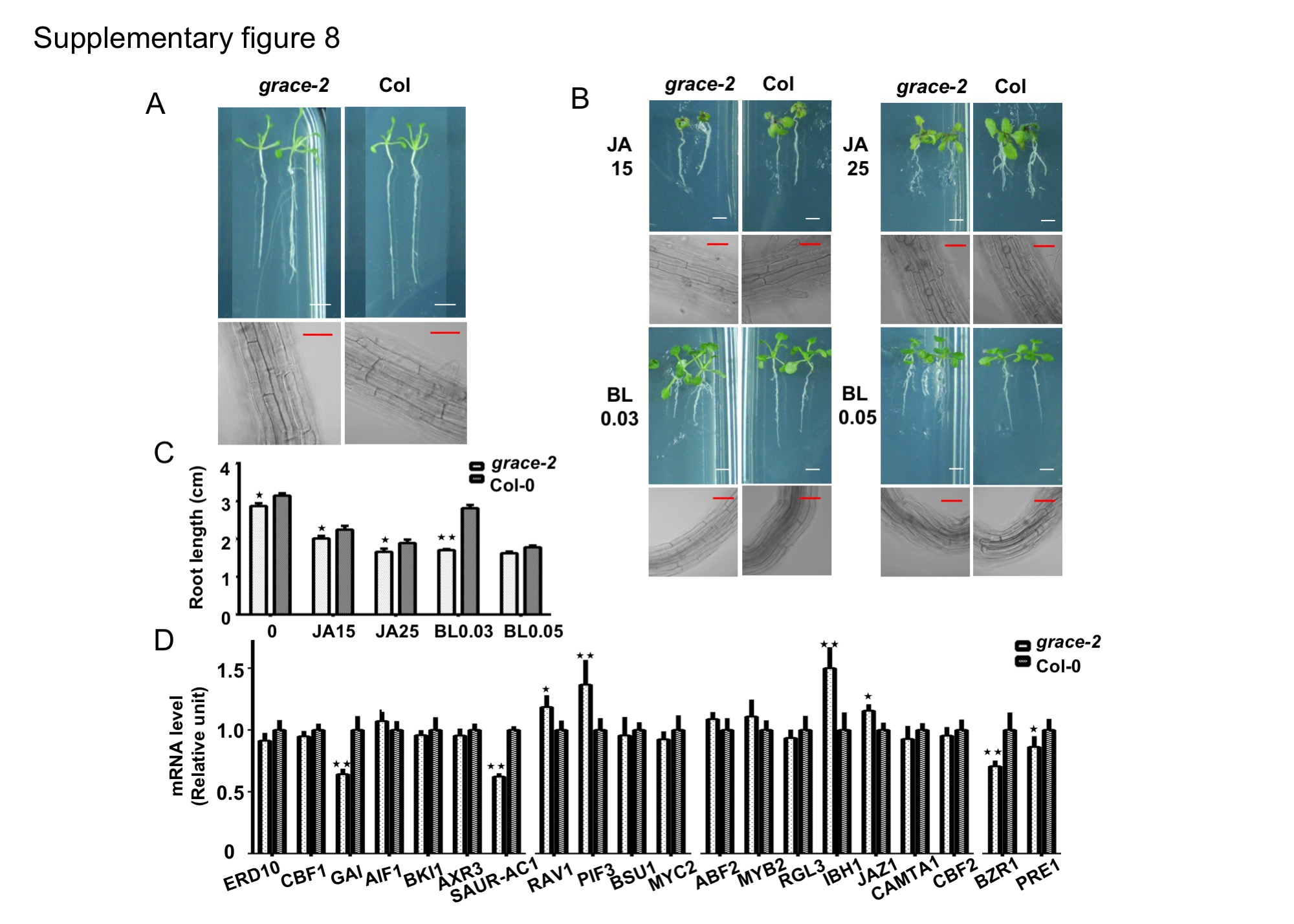

Supplement: Supplementary Figure 8 — Root and root cell of 10-day-old seedlings. (A) Root and root cell of 10-day-old Col-0 and grace-2 without treatment (root, bars, 0.5 cm; root cell, bars, 50 μm). (B) Root and root cell of 10-day-old Col-0 and grace-2 under JA or BL treatment (μM). (C) Statistic analysis of root length of 10-day-old Col-0 and grace-2. (D) Some tested genes were altered in grace-2, compared to Col-0. Each value is the mean ± SE of three determinations. Student's t-test was used to compare the germination rates of each genotype with those of the wild type Col-0 (*P < 0.05, **P < 0.01). [file Image8.JPEG]

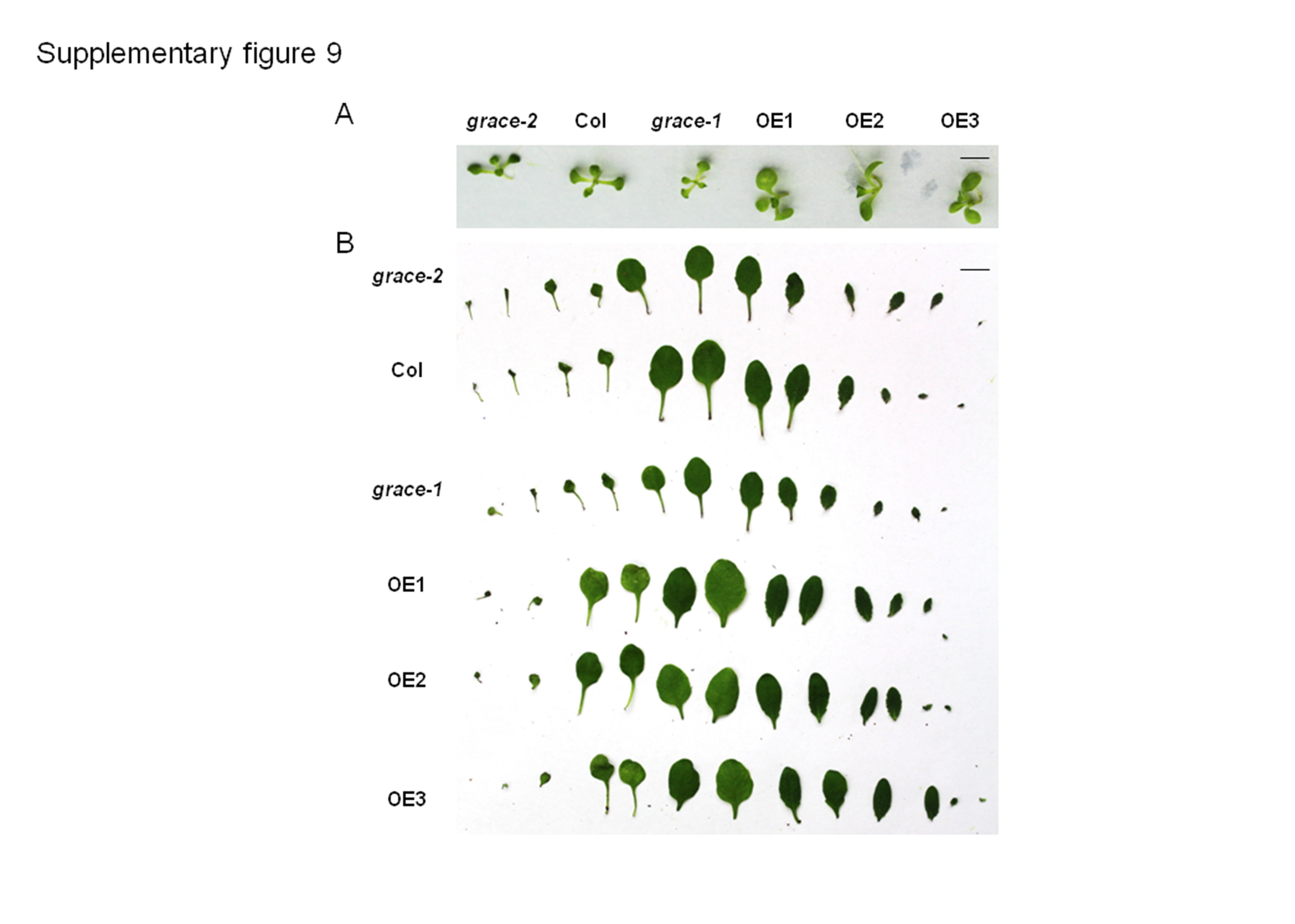

Supplement: Supplementary Figure 9 — Cotyledon and rosette leaf of different genotypes. (A) Cotyledons of 10-day-old seedling (bars, 0.5 cm). (B) Rosette leaf of 4-week-old plant (bars, 1 cm). [file Image9.TIF]
