# Supplementary material for: Functional and Structural Characterization of a Receptor-Like Kinase Involved in Germination and Cell Expansion in Arabidopsis
Source: Front Plant Sci. 2017 Nov 22;8:1999. doi: 10.3389/fpls.2017.01999 (PMC5702872; doi:10.3389/fpls.2017.01999)
Supplement: Supplementary file 10 [file Table1.PDF]

Supplementary table 1 Primers for this study

Gene	Primers for generation of protein expression
GRACE (35-729aa)	forward: 5'- CCCGGGTAAGGATCCGGAGATTCTCTGGACAGTGAT -3'
	reverse: 5'- GAAGTCGACTTAATGATGATGATGATGATGAGTTCTT -3'
	Intermediate primer forward: 5'- GTTCTGCAACAGTTTGGTTGTTGCAAATCTATCGACC -3'
	Intermediate primer reverse: 5'- GGTCGATAGATTTGCAACAACCAAACTGTTGCAGAA -3'
	**Primers for identification of T-DNA insertion mutant**
GRACE (*AT1G74360*)	LB1: 5'- GCCTTTTCAGAAATGGATAAATAGCCTTGCTTCC -3'
	LP-*grace-1*: 5'- TAAGCCATTGAAGAGCTCTGC -3'
	RP-*grace-1*: 5'- TTCCAGTTCAGGTGGATCATC -3'
	LP-*grace-2*: 5'- AACTTCTCCGCTTTAACGGAG -3'
	RP-*grace-2*: 5'- TCAATTTCCCAAATGAAGCTG-3'
	**Primers for generation of overexpressing transgenic plants**
	forward: 5'- GCTCTAGAATGACTATGGTGACGCGTGTG -3'
	reverse: 5'- GGGGTACCCATTTCTATGTAACCTTGTG -3'
	**Primers for quantitative real-time PCR analysis**
Actin2/8 (*AT3G18780*)	forward: 5'- GGTAACATTGTGCTCAGTGGTGG -3'
	reverse: 5'- AACGACCTTAATCTTCATGCTGC -3'
GRACE (*AT1G74360*)	forward: 5'- CTACCCTTCCATCGTTCACAG -3'
	reverse: 5'- CCAAACACTCTTCTCCTCCATC -3'
ABF2 (*AT1G45249*)	forward: 5'- TTGGGGAATGAGCCACCAGGAG -3'
	reverse: 5'- GACCCAAAATCTTTCCCTACAC -3'
AIF1 (*AT3G05800*)	forward: 5'- ATGTCTTCTGAACAAGGAAATGGATC -3'
	reverse: 5'- TTTAGGGAATATACCTTCTGCGCC -3'
AXR3 (*AT1G04250*)	forward: 5'- ATGATGGGCAGTGTCGAGCTGAATC -3'
	reverse: 5'- GCAGGAAACCATCACGTTC -3'
BKI1 (*AT5G42750*)	forward: 5'- TAAGTCATTTTCTCTCTTTGGTTTAT -3'
	reverse: 5'- ACCCATAAGAGAGGAAGAGAAAGAGT -3'
BSU1 (*AT1G03445*)	forward: 5'- GCGGGATTAGAGAAGATGTATTGCTC -3'
	reverse: 5'- AGTGGAACGTATCCTTCAATAGCACTA -3'
BZR1 (*AT1G75080*)	forward: 5'- TCCATGGCCATTGCTAAACAATCAAT -3'
	reverse: 5'- GGTAGGAGAGGTTGGCACCATAGAG -3'
CAMTA1 (*AT5G09410*)	forward: 5'- CTGTCAGAAGCCCAACACAG -3'
	reverse: 5'- CCTTGAGCTTCTCATGAGCTTCTC -3'

CBF1 *(AT4G25490)*	forward: 5’- AACTTCGCTGACTCGGCTTGG -3’
	reverse: 5’- AACAGACGGCGGCGGTAAA -3’
CBF2 *(AT4G25470)*	forward: 5’- CGGTGATTACAGTCCGAAGC -3’
	reverse: 5’- CAAGCCGAGTCAGCGAAA -3’
ERD10 *(AT1G20450)*	forward: 5’- TCTCTGAACCAGAGTCGTTT -3’
	reverse: 5’- CTTCTTCTCACCGTCTTCAC -3’
GAI/RGA2 *(AT1G14920)*	forward: 5’- TTCGGCTTCTTCGTCTAACCAAGGCG -3’
	reverse: 5’- AAGCCAAAAGCGCGTGAACGAGACGC -3’
IBH1 *(AT2G43060)*	forward: 5’- ATGGCCTCTGCAGACAAACTCATAAA -3’
	reverse: 5’- TTACGATCTTGTTGTCATCTTTGTCT -3’
JAZ1 *(AT1G19180)*	forward: 5’- GAGCAAAGGCACCGCTAATA -3’
	reverse: 5’- TGCGATAGTAGCGATGTTGC-3’
MYB2 *(AT2G47190)*	forward: 5’- TGCTCGTTGGAACCACATCG -3’
	reverse: 5’- ACCACCTATTGCCCCAAAGAGA -3’
MYC2 *(AT1G32640)*	forward: 5’- TCATACGACGGTTGCCAGAA -3’
	reverse: 5’- AGCAACGTTTACAAGCTTTGATTG -3’
PIF3 *(AT1G09530)*	forward: 5’- CTCAAAGACGACTATGGTGGACGAGA -3’
	reverse: 5’- ATCCTTTCTTCTCTGAAACAACGGGA -3’
PRE1 *(AT5G39860)*	forward: 5’- ATGTCGAACAGAAGATCAAGGCAATC -3’
	reverse: 5’- TTACATGAGTAGGCTTCTAATAACGGCGG -3’
RAV1 *(AT1G13260)*	forward: 5’- TGTACATTGGGTGGAAGTCGAG -3’
	reverse: 5’- TTACGAGGCGTGAAAGAT -3’
RGL3 *(AT5G17490)*	forward: 5’- TGCCTCTTCTAGTAATGCCTTCAATGA -3’
	reverse: 5’- CGAGGCTCAGATTCTCCAGCTGAACC -3’
SAUR-AC1 *(AT4G38850)*	forward: 5’- ATGGCTTTTTTGAGGAGTTTCTTGG -3’
	reverse: 5’- TGTGAAGAACAAAGATTCATGACATGGT -3’
