# Supplementary material for: Functional and Structural Characterization of a Receptor-Like Kinase Involved in Germination and Cell Expansion in Arabidopsis
Source: Front Plant Sci. 2017 Nov 22;8:1999. doi: 10.3389/fpls.2017.01999 (PMC5702872; doi:10.3389/fpls.2017.01999)
Supplement: Supplementary file 11 [file Table2.PDF]

**Supplementary table 2** Data collection and refinement statistics

| | |
|---|---|
| Data collection statistics | |
| Beam Line | Beam line BL17U1, SSRF |
| Space Group | $P2_12_12_1$ |
| Number of reflections | 42614 (3818) |
| Cell dimensions | |
| *a, b, c* (Å) | 59.583, 155.286, 190.213 |
| *α, β, γ* (°) | 90.00, 90.00, 90.00 |
| Resolution (Å) | 50.00-2.83（2.90-2.83） |
| $R_{sym}$ (%) | 7.2 (43.3) |
| $I/\sigma (I)$ | 12.1 (9.6) |
| Completeness (%) | 98.6 (84.0) |
| Redundancy | 3.6 (3.7) |
| Refinement Statistics | |
| Resolution (Å) | 50.00-2.83（2.90-2.83） |
| Number of reflections | |
| $R_{work}/R_{free}$ (%) | 22.6/28.9 |
| Number of molecules/ asymmetric unit r.m.s deviations | 2 |
| Bond lengths (Å) | 0.01 |
| Bond angles (°) | 1.57 |
| Ramachandran (%) (from PHENIX) | |
| Preferred region | 84.4 |
| Allowed region | 12.5 |
| Outliers | 3.1 |
